# Processed Food as a Risk Factor for the Development and Perpetuation of Crohn’s Disease—The ENIGMA Study

**DOI:** 10.3390/nu14173627

**Published:** 2022-09-02

**Authors:** Gina L. Trakman, Winnie Y. Y. Lin, Amy L. Hamilton, Amy L. Wilson-O’Brien, Annalise Stanley, Jessica Y. Ching, Jun Yu, Joyce W. Y. Mak, Yang Sun, Junkun Niu, Yinglei Miao, Xiaoqing Lin, Rui Feng, Minhu Chen, Nitin Shivappa, James R. Hebert, Mark Morrison, Siew C. Ng, Michael A Kamm

**Affiliations:** 1 Department of Gastroenterology, St Vincent’s Hospital, Melbourne 3065, Australia; 2 Department of Medicine, The University of Melbourne, Melbourne 3065, Australia; 3 Department of Dietetics, Nutrition and Sport, La Trobe University, Melbourne 3086, Australia; 4 Department of Medicine and Therapeutics, The Chinese University of Hong Kong, Hong Kong, China; 5 Microbiota I-Center (MagIC), The Chinese University of Hong Kong, Hong Kong, China; 6 Institute of Digestive Disease, State Key Laboratory of Digestive Diseases, The Chinese University of Hong Kong, Hong Kong, China; 7 Department of Gastroenterology, The First Affiliated Hospital of Kunming Medical University, Kunming 650032, China; 8 Yunnan Province Clinical Research Center for Digestive Diseases, Kunming 650032, China; 9 Department of Gastroenterology, The First Affiliated Hospital, Sun Yat-sen University, Guangzhou 510060, China; 10 The Cancer Prevention and Control Program, Department of Epidemiology and Biostatistics, Norman J Arnold School of Public Health, University of South Carolina, Columbia, SC 29208, USA; 11 Department of Nutrition, Connecting Health Innovations LLC, Columbia, SC 29201, USA; 12 The University of Queensland Diamantina Institute, Faculty of Medicine, Translational Research Institute, Brisbane 4102, Australia; 13 Centre for Gut Microbiota Research, The Chinese University of Hong Kong, Hong Kong, China

**Keywords:** inflammatory bowel disease, Asia, nutrition, dietary inflammatory index, diet pattern

## Abstract

(1) Background: Developing countries have experienced a rapid recent rise in Inflammatory Bowel Disease (IBD) incidence and emerging evidence suggests processed foods and food additives may predispose one to the development and perpetuation of Crohn’s disease (CD). The aim of this study was to evaluate processed food and food additive intake in CD patients and controls, in Australia (high CD incidence), Hong Kong (intermediate incidence) and mainland China (emerging incidence). (2) Methods: In 274 CD patients (CD), 82 first-degree relatives (FDR), 83 household members (HM) and 92 healthy unrelated controls (HC) from Australia (n = 180), Hong Kong (HK) (n = 160) and mainland China (n = 191) we estimated early life (0–18 years), recent (12 months), and current processed and food additive intake, using validated questionnaires and a 3-day-food diary. (3) Results: Early life processed food intake: Combining all regions, CD were more likely to have consumed soft drinks and fast foods than HM, more likely to have consumed processed fruit and snacks than their FDR, and more likely to have consumed a range of processed foods than HC. HK and China CD patients were more likely to have consumed a range of processed foods than HC. Recent food-additive intake (12-months): Combining all regions, CD patients had significantly higher intakes of aspartame and sucralose, and polysorbate-80, than HC, and more total emulsifiers, artificial sweeteners, and titanium dioxide than FDR and HC. HK and China CD patients had a higher intake of almost all food additives than all controls. Current additive intake (3-days): Australian and HK CD patients had higher total food-additive intake than FDR, and HK CD patients had a higher intake of total food-additives and emulsifiers than HM. (4) Conclusions: CD patients have been exposed to more processed food and food additives than control groups, which may predispose them to CD development and ongoing inflammation.

## 1. Introduction

The pathogenesis of Crohn’s disease is being unraveled. Genetic predisposition is a key factor in some patients but is not always present, and predominantly mediates risk through an effect on enteric bacterial handling [1]. Environment factors, including enteric microbial homeostasis, are believed to play a central pathogenic role [2,3,4]. 

Developed industrialized nations, which have a high Crohn’s disease incidence [5], are characterised by high exposure to many putative environmental risk factors, including early life exposure to antibiotics and ultra-processed food [6,7]. Developing countries have experienced a rapid recent rise in IBD incidence, likely related to changes in environmental exposure [4,8]. Diet is a key environmental factor that changes with industrialization. Epidemiological studies and controlled trials suggest that diet affects Crohn’s disease risk and disease activity [7]. Laboratory studies provide further biologically plausible mechanisms by which food, nutrients and additives can have pathologic or protective effects, with the gut microbiome playing a significant role [9]. The exact food components that play a role, and whether there are differences in regions of established and emerging disease, remain to be determined.

Recent meta-analyses [10,11] and cohort studies [12,13,14] have assessed the relationship between dietary patterns and the development of Crohn’s disease, with both positive [10] and no association [12] to a Western diet reported. Variable associations have also been reported in relation to Crohn’s disease risk and “healthy” diets [12], Mediterranean diets [13], and traditional French diets [12]. Diets have recently been characterised as having “inflammatory potential”, and such empirical dietary inflammatory potential (EDIP) has been associated with an increased Crohn’s disease risk [14]. Recently published evidence links diet-associated inflammation, as quantified using the dietary inflammatory index (DII^®^) [15], both to diseases of the gastrointestinal tract [16,17,18], and to consumption of ultra-processed foods [19].

A review of meta-analyses that focused on single nutrients and foods found that fruit and fibre intake decreased Crohn’s risk, while sucrose increased that risk [20]. Other dietary factors have been associated with decreased (vegetables, fish) or increased (red meat, processed meat and fat) Crohn’s disease risk [21,22].

Food additives have recently emerged as potential central factors causing a microbial change that drives inflammation. Emulsifiers and nanoparticles are the major additives evaluated to date [23,24,25,26,27]. Emulsifiers are ubiquitous in ultra-processed foods and are used to homogenise foods, extend their shelf life, and improve food texture [24]. Emulsifiers change the microbial population, resulting in the degradation of the gut mucous layer and ingress of bacteria into the epithelium [23,24,25]. Titanium dioxide and aluminosilicate microparticles may promote immunogenic responses in gastrointestinal mucosal lymphocytes [27] and perpetuate low-grade inflammatory responses via intracellular uptake and accumulation of particles within the gut [26]. Several other additives have also been linked to Crohn’s disease. Artificial sweeteners such as sucralose and saccharin promote the growth of pro-inflammatory *Proteobacteria*, purported to be important in Crohn’s disease [28]. Sulphites disturb mucosal integrity and impair butyrate oxidation, leading to inflammation in colonic cells [29].

This study aimed to determine if food and food additive intake differed in Crohn’s disease patients compared to relevant control (related, cohabiting, and healthy) groups. Attention has been given to early childhood, the key period of microbiota formation and determinant of Crohn’s disease risk [30], and to current intake in relation to ongoing disease activity. Attention has also been given to evaluating these dietary factors in different geographical regions which represent populations with high (Australia), intermediate (Hong Kong) and emerging (mainland China) Crohn’s disease incidence [5,31,32].

## 2. Materials and Methods

### 2.1. Study Design and Participants

This case–control, cross-sectional, observational study assessed four participant groups: patients with Crohn’s disease, their first-degree relatives, their household members, and non-cohabiting healthy unrelated controls, across three regions: Australia, Hong Kong, and China. Retrospective data on early life and recent (last 12 months) diet intake was collected at a single time point from all participants. Current dietary intake data (prospective 3-day food diaries) was collected from participants in Australia and Hong Kong; current dietary data was not collected from participants in China for technical reasons.

#### 2.1.1. Inclusion and Exclusion Criteria

Participants were included if they were aged 18 years or older, able to provide informed consent, and lived in the same geographic area for the preceding 6 months. Crohn’s disease had been confirmed using clinical, endoscopic, and histologic criteria.

Exclusion criteria were use of prebiotics, probiotics, antibiotics, laxatives or anti-diarrheal medication in the last 3 months, recent dietary change, known current complex infection or sepsis, history of severe organ failure, bowel surgery in the last 6 months, presence of a bowel stoma, current pregnancy, and colonoscopy in the last month.

#### 2.1.2. Enrolment

Participants were enrolled between October 2018 and December 2019 from outpatient clinics at secondary and tertiary hospitals. First-degree relatives and household members were approached to participate in the outpatient clinic or by telephone. Healthy unrelated controls were identified from colonoscopy screening lists (Australia, Hong Kong, China), or from the general population by advertisement (Hong Kong, China). Where colonoscopy screening lists were used, participants were excluded if polyps or other gastrointestinal disease were identified.

The study procedure was approved by local ethics committee of each hospital and all participants gave informed written consent.

#### 2.1.3. Demographic and Clinical Information

Demographic and clinical information were obtained by interview and electronic medical record review. Crohn’s disease phenotype, duration, and activity, based on the Crohn’s Disease Activity Index (CDAI), were recorded.

### 2.2. Food Intake Questionnaires

Participants were given a set of validated dietary questionnaires and food record templates to self-report dietary intake, from which we derived data on food additive and nutrient intake. The dietary questionnaires have been validated in a separate study, using a different sample derived from the general population. Participants in both the validation study and the current study were provided with content explanation, time to check with family members about early life food intake, then returned questionnaires at their research visit about one week later. Questionnaires were then checked for participant understanding and completeness, and missing data entered by discussion with the participant.

#### 2.2.1. Early Life Processed Food Intake

This was assessed using a previously validated questionnaire [33]. This multiple-choice questionnaire comprises 42 items about food habits and specific food consumption, in specific age ranges from infancy to the age of 18 years: 4–12 months, 1–5 years, 5–10 years, and 10–18 years.

#### 2.2.2. Recent (Last 12 Months) Food Additive Intake

This was assessed using a previously validated [33] food frequency questionnaire. The questionnaire addresses intake frequency and amount over the past 12 months. Maximal exposure to food additives (aluminosilicates, aspartame, carboxymethylcellulose, carrageenan, polysorbate-80, saccharin, sucralose, sulphites, titanium dioxide) was then estimated in mg/year based on the maximal permissible level (MPL) of additives in the food, or concentration data from the literature.

#### 2.2.3. Current (Last 3-Days) Food Additive and Nutrient Intake

Current food additive and nutrient intake were assessed using a prospective 3-day food record. The food record was checked for completeness and accuracy in an interview with qualified dietitian. Assessment of additive intake from 3-day food diaries was estimated using methods previously outlined [33]. These data were obtained for Australia and Hong Kong only. Healthy controls, who were undergoing colonoscopy, were instructed to complete their food diaries before starting colonoscopy preparation.

DII scores were calculated based on the average of the 3-day food diaries, which included supplements, using methods spelled out previously [15]. A total of 24 food parameters (of a possible 45) were included. For most analyses, DII was reported in absolute terms, not adjusted for energy intake. Where additives were reported relative to body weight (mg/kg) the energy-adjusted DII was used.

#### 2.2.4. Nutrient Database Harmonization

Qualified dieticians entered food data into FoodWorks version 10 (Xyris, Brisbane, QLD, Australia) for Australian data and Food Processor Nutrition Analysis version 11 (ESHA, Salem, OR, USA) for Hong Kong and mainland China data. Different, locally validated nutrient databases were used for each region. Where energy and nutrients differed in FoodWorks and ESHA databases data harmonization was undertaken.

### 2.3. Statistical Methods

The study population comprised 274 Crohn’s disease patients and 257 control subjects. When comparing total yearly additive intake of all Crohn’s disease patients to total yearly additive intake of all control subjects (all regions combined), a sample size of 100 participants is required to achieve 80% power to show a difference at an alpha value of 0.05 using a one tailed *t*-test.

#### Data Analyses

Data were analyzed using SPSS version 25 software (IBM Corp., Armonk, NY, USA). Data may be made available on reasonable request. Weekly consumption of ultra-processed and processed food in early life are reported as proportions. Normality of continuous variables (additive intake, DII) were assessed by the Shapiro–Wilk test, where a non-statistically significant value indicates data are normally distributed. All additive data were non-normal and are reported as median [interquartile range].

Early life processed food, recent and current additive intake, and DII were analyzed to ascertain differences across regions and between Crohn’s disease and all controls combined, first-degree relatives, household members, and healthy control groups. Comparisons between Crohn’s disease and controls were conducted with all regions combined as well as within individual regions (Supplementary Table S1). When a first-degree relative was also a household member they were included in the analyses in both participant groups; this included a total of 34 participants (17 in Australia, 17 in Hong Kong).

Comparisons across regions were made using Chi-squared analysis for categorical variables and Kruskal–Wallis for continuous variables. Where differences were statistically significant for categorical, continuous variables, additional pair-wise Chi-squared or Mann–Whitney U tests, respectively, were conducted to determine which groups differed.

Crohn’s disease patients were compared with matched first-degree relatives and household members using McNemar’s test for categorical variables or Wilcoxon (symmetrical differences between groups) or Sign rank test (asymmetrical differences between groups) for continuous variables. Crohn’s disease patients were compared with healthy controls, unmatched household members, first-degree relatives, using Chi-square analysis for categorical variables or Mann–Whitney U test for continuous variables.

The relationships between energy-adjusted DII and additive intake (mg/per kg body weight) was assessed using Spearman’s rank correlation coefficient. The relationships between CRP, hemoglobin, CDAI, versus current additive intake and DII were assessed using Spearman’s rank correlation coefficient; values of 0.3, 0.5 and 0.8 representing weak, moderate and strong correlations, respectively. Given that dietary additives are likely to influence Crohn’s disease activity via the gut microbiome, it is likely that total exposure to additive intake is the variable of importance. Therefore, when assessing additive intake from 3-day food records, we did not correct for total energy intake because we were interested in absolute, not relative, additive consumption.

## 3. Results

### 3.1. Participant Characteristics

957 participants were screened for inclusion in the study; 411 participants did not meet eligiblity criteria or were not interested in participating in the study and 15 participants were withdrawn from study. A total of 531 participants (274 Crohn’s disease and 257 controls) were eligible for inclusion (Supplementary Figure S1). The median age of participants was 43 years, 53% were male, median weight was 65 kg, and BMI 23 kg/m^2^. Baseline characteristics are described in Table 1.

All participants (100%) in Mainland China reported their ethnicity as Mainland Chinese. Participants in Hong Kong were 91.8% Hong Kong Chinese and 8.2% Mainland Chinese. Participants in Australia were predominately Caucasian (73.5%), with the remainder of participants reporting their ethnicity as ‘Other’(20.0%), Jewish (5.9%) and HK Chinese (0.6%). Of those who classified their ethnicity as ‘Other’, 1.6% stated they were Chinese, 0.6% Eurasian, and 0.6% Vietnamese. The remainder of the participants were Caucasian European (10.3%), South Asian (3.2%), African (1.2%), Middle Eastern (0.6%), Aboriginal (0.6%), or Pacific Islander (0.6%), with 1.2% choosing ‘prefer not to say’.

Participants with Crohn’s disease were predominately (85%) in remission with a median CDAI of 64 (Table 2).

### 3.2. Food Intake

A summary of all significant results is provided in Table 3.

#### 3.2.1. Early Life Intake—Infancy to 18 years

Definitions of processed food are included in Supplementary Table S2. The proportion of participants who consumed ultra-processed and processed foods on a weekly basis and *p*-values are outlined in Supplementary Table S3.

##### Household Members

When combining all regions, Crohn’s disease patients had a significantly higher intake of soft drinks (aged 4–12 months) and fast food (aged 10–18 years) than matched household controls. When addressing individual regions, Australian Crohn’s disease patients had a significantly higher rate of fast-food intake when aged 5–10 years than matched household controls.

##### First-Degree Relatives

When combining all regions, Crohn’s disease patients had a significantly higher intake of processed fruit (aged 4–12 months) and ultra-processed snacks (aged 4–12 months) than matched first-degree relatives.

##### Healthy Unrelated Controls

Across all the regions, Crohn’s disease patients had more frequent processed meat, processed grains, fast food, soft drinks, and ultra-processed snack intake, in all age groups except 4–12 months, than healthy unrelated controls. Crohn’s disease patients had more frequent intake of processed fruit than healthy unrelated controls in all age groups. Crohn’s disease patients had less frequent intake of processed vegetables than healthy unrelated controls in all age groups.

In Hong Kong, Crohn’s disease patients had significantly less frequent intake of ultra-processed dairy at all ages apart from 1–5 years, and significantly more frequent intake of fast food (aged 1–5 years and 5–10 years), soft drink (aged 5–10 years), and ultra-processed snacks (aged 5–10 years), than healthy unrelated controls.

In China, Crohn’s disease patients had significantly more frequent intakes of ultra-processed dairy (aged 5–10 years), processed meat (aged 1–5 years and 10–18 years), processed grains (all age groups except 4–12 months), soft drink (aged 5–10 years and 10–18 years), and ultra-processed snacks (all age groups except 4–12 months) than healthy unrelated controls. In China, Crohn’s disease patients had significantly less frequent intake of processed vegetables, including fermented vegetables, in all age groups except 10–18 years, than healthy unrelated controls.

##### Regional Differences

Almost all food variables and breastfeeding differed significantly for all age groups between the three regions (Supplementary Table S3). The rate of breastfeeding and consumption of home-grown foods were highest in mainland China, followed by Australia and lowest in Hong Kong. Intake of frozen, canned, or pickled vegetables was highest in mainland China, followed by Australia and lowest in Hong Kong. Most other ultra-processed and processed foods (fruits, grains, snacks, soft drink, dairy, and fast food) consumption was lower in mainland China than in both Hong Kong and Australia. Processed meat and processed grain intakes were higher in Hong Kong than Australia, and soft drink, processed dairy and fast-food intakes were higher in Hong Kong than in Australia.

#### 3.2.2. Recent—Past 12 Months Additive Intake

Median recent additive intake and specific *p*-values are described in Supplementary Table S4.

##### Household Members

When combining all regions Crohn’s disease patients consumed significantly more polysorbate-80 than their matched household members. Crohn’s disease patients in Hong Kong had a higher intake of total additives, aspartame, and total artificial sweeteners than matched household members. Crohn’s disease patients in China consumed significantly more polysorbate 80, carboxymethylcellulose, carrageen, and total emulsifiers than their matched household members.

##### First-Degree Relatives

When combining all regions Crohn’s disease patients consumed significantly more polysorbate-80, carrageenan, carboxymethylcellulose, aluminosilicates, titanium dioxide, sucralose, total emulsifiers, and total additives than their first-degree relatives.

In Australia, Crohn’s disease patients had a significantly higher intake of saccharin, polysorbate-80, total emulsifiers, and titanium dioxide than their matched first-degree relatives.

In Hong Kong Crohn’s disease patients had a higher intake of polysorbate-80, carboxymethylcellulose, total artificial sweetener, aspartame, sucralose, saccharin intake, titanium dioxide, and aluminosilicates than their matched first-degree relatives.

In mainland China, Crohn’s disease patients had a significantly higher intake of aluminosilicates but lower intake of sulphites than their matched first-degree relatives.

##### Healthy Unrelated Controls

When combining all regions Crohn’s disease patients consumed significantly more of all additives than healthy unrelated controls. Crohn’s disease patients in Australia had a significantly higher intake of polysorbate-80, and Hong Kong Crohn’s patients had a significantly higher intake of titanium dioxide, than healthy unrelated controls.

In China, Crohn’s disease patients had a significantly higher intake of carrageenan and aluminosilicates, but significantly lower intake of titanium dioxide, than healthy unrelated controls.

Comparisons between Crohn’s disease participants and all controls combined are provided as Supplementary Material (Supplementary Table S4).

##### Regional Differences

**Figure 1:**
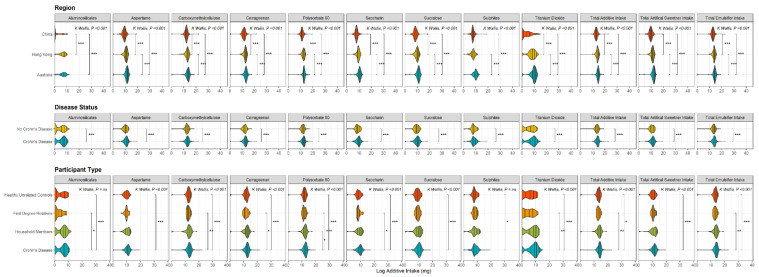
Violin plots showing the distribution of Log-adjusted median additive intake (in milligrams) across additives of interest (as well as total additive intake, total artificial sweetener intake and total emulsifier intake, far right panels) from recent (last 12 months) intake questionnaire, between regions (top panel), for Crohn’s cases compared to all controls (Disease status; middle panel), and between all participant types (bottom panel). Lines represent quantiles (0.25, 0.50, 0.75, 0.95), all *p* values calculated on unadjusted intake amounts, only significant *p* values shown (* *p* ≤ 0.05, ** *p* ≤ 0.01, *** *p* < 0.001, ns—not significant Comparisons of ≥3 groups performed with Kruskal–Wallis test, between group comparisons calculated with Mann–Whitney U or Wilcoxon sign-rank test.

The nature of diet content differed between Australia and Hong Kong. Intake of all additives except aluminosilicates were statistically significantly higher in Australia than Hong Kong. Intake of all additives were higher in Australia than in China. Intake of all additives except sulphites were higher than in Hong Kong than in China (Figure 1, Supplementary Table S4).

#### 3.2.3. Current—3 Days Additive Intake

**Figure 2:**
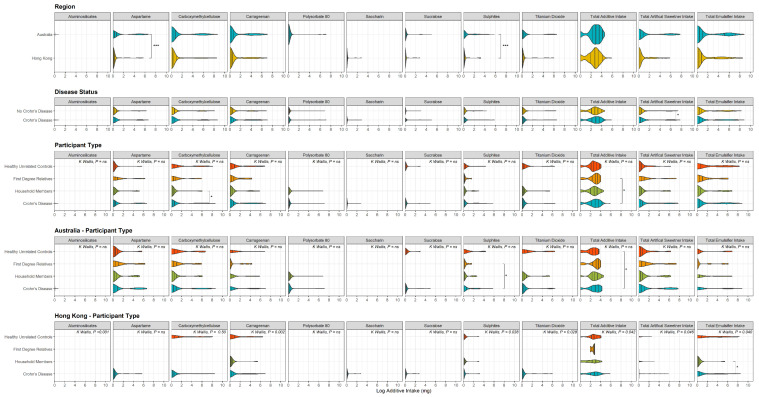
Violin plots showing the distribution of Log-adjusted median additive intake (in milligrams) across additives of interest (as well as total additive intake, total artificial sweetener intake and total emulsifier intake, far right panels) from current (last 3 days) intake questionnaire: between regions (top panel), for Crohn’s cases compared to all controls (Disease Status; second panel), between all participant types across both regions (third panel), and between all participant types with each region (bottom two panels). Lines represent quantiles (0.25,0.50, 0.75, 0.95), all P values calculated on unadjusted intake amounts, only significant P values shown (* *p* ≤ 0.05, *** *p* < 0.001, ns—not significant). Comparisons of ≥3 groups performed with Kruskal–Wallis test, between group comparisons calculated with Mann–Whitney U or Wilcoxon sign-rank test.

Median current additive intakes are described in Figure 2 and Supplementary Table S5.

##### Crohn’s Disease Patients Compared to Controls

When combining Australia and Hong Kong, Crohn’s disease patients had a significantly higher intake of carboxymethylcellulose than matched household members (*p* = 0.024), and significantly more total additives than their matched first-degree relatives (*p* = 0.04) and healthy unrelated controls (*p* = 0.042).

In Crohn’s disease patients in Hong Kong total emulsifier intake was higher than in matched household members (*p* = 0.04).

In Australia, Crohn’s disease patients’ sulphite intake (*p* = 0.02) was significantly higher than their matched first-degree relatives.

##### Regional Differences

When combining all subjects within each of Australia and Hong Kong the intake of aspartame and sulphites was significantly higher in Australia than in Hong Kong. 

### 3.3. Dietary Inflammatory Index (DII)

DII, energy, and nutrient intake are described in Supplementary Table S6.

#### 3.3.1. Crohn’s Disease Patients Compared to Controls

When combining all regions, Crohn’s disease patients had a significantly higher DII, indicative of a more pro-inflammatory diet, than all controls combined (1.4453 vs. 0.7582, *p* = 0.011).

In Hong Kong, Crohn’s disease patients had a higher DII (2.7051) than their matched household members (2.204) (*p* < 0.001)

When combining Australia and Hong Kong, and within Hong Kong alone, Crohn’s disease patients had a significantly higher DII than their matched first-degree relatives (Combined: 1.44 vs. 0.56, *p* = 0.001); Hong Kong: 2.75 vs. 2.28, *p* = 0.008)

#### 3.3.2. Regional Differences

When combining all subjects within each of Australia and Hong Kong the DII was significantly lower in Australia (−0.22) compared to Hong Kong (2.60) (*p* < 0.001).

##### Relationship between DII and Total Additive Intake

There was a significant but weak correlation between DII and total additive intake, when all subjects were considered (r = 0.145, *p* = 0.015), and across all Australian (r = 0.170, *p* = 0.032) and all Hong Kong subjects (r = 0.191, *p* = 0.035).

When combing Crohn’s disease subjects in Australia and Hong Kong (r = 0.198, *p* = 0.012), and within Australian Crohn’s disease patients (r = 0.261, *p* = 0.013), there was a significant but weak correlation between DII and total additive intake. For Hong Kong Crohn’s disease patients, the correlation was not significant (r = 0.221, *p* = 0.060) (Supplementary Table S7).

### 3.4. Disease Activity and Dietary Intake

Disease diagnosis and disease activity details are shown in Table 2. Seventy percent, 75% and 67% of Crohn’s disease patients in Australia and Hong Kong combined, Australia alone, and Hong Kong alone, respectively, had a CRP within the normal range (<5 mg/L). When Australia and Hong Kong patients were considered together, there was a weak, positive correlation between the DII and CRP (r = 0.244, *p* < 0.001).

Correlations between nutrient intake and CRP, Hemoglobin, and CDAI are shown in Supplementary Table S7.

Correlation between energy-adjusted DII and current, total additive intake (mg/per kg body weight) are shown in Supplementary Table S8. There was a weak, positive correlation between energy-adjusted DII and total additive intake (mg/kg body weight/day) across the whole cohort (r = 0.144, *p* = 0.015), in all Australian participants (r = 0.019, *p* = 0.032), in all Hong Kong participants (r = 0.198, *p* = 0.012), in Australian Crohn’s disease participants (r = 0.261, *p* = 0.013), and in all Crohn’s participants combined (r = 0.198, *p* = 0.12).

There were no significant differences in DII or nutrient intake on the basis CRP or Hemoglobin range (Supplementary Table S9).

## 4. Discussion

Food is increasingly considered to play a role in the aetiology and pathophysiology of Crohn’s disease, with ultra-processed and processed foods and dietary additives thought to mediate increased disease risk via perturbation of gut microbiota homeostasis [9,34].

### 4.1. Early Life Processed Food Intake

The key finding in this cross-cultural and geographically diverse study is that Crohn’s disease patients in early life had an increased intake of ultra-processed and processed foods compared to various control groups. This overall excess ultra-processed and processed food intake was true in relation to healthy unrelated controls, first-degree relatives (who might be considered to share genetic risk), and household controls (who share current risk factors). First-degree relatives included both siblings and parent/children pairings, with some first-degree relatives also being current household members. Similar food intake between siblings (compared to parents and children) is expected in early life, with current intake more likely to be similar between household members. However, household members do not always consume identical diets, especially since it is common to consume meals out of the home.

The key findings support the notion that dietary factors, especially pro-inflammatory ultra-processed food containing food additives, in early life may be a key risk for the later development of Crohn’s disease. These data on ultra-processed food exposure in early life complement the finding that early life exposure to antibiotics, a potent cause of microbiome changes, increases the risk of developing Crohn’s disease [35]. Just as there are different foods and food additives which are likely to vary in the risk they bestow on changing the microbiome and enhancing Crohn’s disease risk, there are different types of antibiotics which are likely to vary in the risk they bestow for developing Crohn’s disease. Total ultra-processed food and food additives can be considered in the same way as total antibiotic exposure, with the risk associated with key individual food additives or individual antibiotics remaining to be determined.

The extent of ultra-processed and processed food, dietary additives consumption varied across the three regions. This has the most relevance in terms of the whole population risk for developing Crohn’s disease, and for its perpetuation once established. The former was roughly proportional to the incidence of Crohn’s disease in the three regions with low, medium, and high population intake associated with low, medium and high Crohn’s disease incidence, in mainland China, Hong Kong and Australia respectively. Similarly, in data from the Prospective Urban Rural Epidemiology (PURE) cohort, Narula et al. [36] found higher ultra-processed food intake in North America, Europe, and South America compared to Africa, the Middle East, South Asia, South East Asia and China.

Fast food intake in early life was frequent in the Australia and Hong Kong populations. This concurs with a 2017–2018 survey that found that 57 percent of Australians eat fast-food once per week or more, with 55% of Australians in our survey reporting weekly fast-food consumption aged 10–18 years [37]. When assessing all regions combined, frequency of fast-food intake in early life was even higher in Crohn’s disease patients than all controls combined.

In a previous, preliminary study we found that patients with inflammatory bowel disease in Australia and Hong Kong reported more frequent intake of fast-food during childhood than healthy controls [38]. Similarly, in an Australian cohort, Niewiadomski et al. [39] found fast-food consumption before the age of 20 more than doubled the risk of developing Crohn’s disease. In a Swedish case–control study, Persson et al. [40] found that consuming fast-food two times per week, in the preceding 5 years, was associated with a three times or greater risk of developing of Crohn’s disease.

Fast-food often contains food additives. Although the emulsifier polysorbate-80 has been described as ‘ubiquitous’ in the food supply, we found it to be present only in some fast foods. Polysorbate-80 has been shown to alter microbial composition, leading to epithelial encroachment and inflammation [23]. Work from our own group has also demonstrated that polysorbate-80 has a bacteriostatic effect of on *Faecalibacterium prausnitzii*, an anti-inflammatory species whose abundance is reduced in Crohn’s disease patients [41].

Some processed food may be protective for the development of Crohn’s disease. Processed vegetable (e.g., pickled and fermented vegetables) intake was very high in mainland China in all subjects, and in all regions was numerically higher in non-Crohn’s disease subjects than Crohn’s disease patients. Although processed vegetables can contain sulphite preservatives and other additives, they also include fermented vegetables. Pao Cai is a spontaneously fermented cabbage eaten in mainland China, however impact of Pao Cai on the gut microbiota and general health has not been well studied. Nevertheless, Pao Cai has been shown to have a predominance of beneficial *Lactobacillus* species [42] and microorganism extracted from Pao Cai have been associated with a number of health benefits in animal models [43,44]. Given the abundance of *Lactobacillus* species, Pao Cai may be comparable to Kimchi, a fermented vegetable dish from Korea. Kimchi has been shown to increase abundance of anti-inflammatory bacterium and modify gene expression in humans, and bacterial strains found in Kimchi have been shown to decrease inflammatory activity of lamina propria lymphocytes in animal models [45]. More generally, healthy individuals following a diet high in fermented foods (including vegetables, kombucha, and yoghurt) have been shown to exhibit a decrease in inflammatory markers and increased microbial diversity [46,47].

Immigration studies have highlighted that childhood is the critical time for exposure to environmental risk factors to impact on the development of inflammatory bowel disease [48,49]. It is therefore not surprising that contemporaneous adult studies have a lower chance of identifying processed foods having an association with the development of new incident IBD. Based on data from NutriNet- Santè cohort Vasseur et al. [12] documented the current diet and followed an adult cohort for a mean follow up of 2.3 years. No dietary patterns or the ultra-processed food proportion in the diet were significantly associated with the risk of incident inflammatory bowel disease. In contrast Lo et al. [50] studied dietary intake over a much longer period in participants of the Nurses’ Health Study (1986–2014), the Nurses’ Health Study II (1991–2017), and the Health Professionals Follow-up Study (1986–2012). Secondary analyses of dietary data found that ultra-processed food consumption was associated with an increased risk of developing incident Crohn’s disease but not ulcerative colitis. A retrospective review of the abovementioned PURE cohort evaluated the relationship between processed food intake and the risk of developing inflammatory bowel disease [36]. In 116,087 adults, enrolled between the ages of 35 to 70, from 21 countries followed for a median of 9.7 years, 90 subjects developed Crohn’s disease and 377 developed ulcerative colitis. Processed soft drinks, sweets, salty snacks, and processed meat were associated with a higher risk for the development of inflammatory bowel disease. Notably, these are the same processed foods identified in the current study when combining all regions and comparing Crohn’s disease patients to unrelated controls, although we classified sweets and salty snacks as a single group (processed snacks).

### 4.2. Recent and Current Food Additive Intake

In our study, recent and current food intake were considered as risk factors for perpetuating existing disease and inflammation. Usual (12 month) intake of most food additives was higher in Crohn’s disease patients than controls in the three studied populations. Current (3 day) intake of select additives (carboxymethylcellulose, sulphites) was also higher in Crohn’s disease patients than controls. There is a biologically plausible explanation for the association between these additives and inflammatory bowel disease. Carboxymethylcellulose is an emulsifier commonly used in bakery products, cordials, ice-creams, dairy drinks, coconut milk, dips, dressings, sauces, and vegan or vegetarian products. In susceptible mice, carboxymethylcellulose has been shown to increase bacterial overgrowth and bacterial adherence to the mucosa [51]. Sulphites are commonly used as dietary additives (e.g., sodium sulphite E221) to preserve food such as meat, wine and beer, dried fruit, cordial, seafood, and biscuits. Sulphate is the main oxidation product of sulphite. Sulphate-reducing bacteria use sulfur and sulphate as energy sources and thereby produce hydrogen sulphide, which can break di-sulphide bonds in the mucus layer of the gut, effecting the integrity of the gut epithelium [52,53].

There are several ways to quantitate estimates of food additive intake. At a whole population level, methods include theoretical food consumption data and Maximal Permissible Level for the additive (Tier 1 Estimates), actual national food consumption data and Maximal Permissible Level for the additive (Tier 2 estimates) and actual national food consumption data and Maximal Permissible Level for the additive and actual use levels of the additive (Tier 3 estimates) [54]. To our knowledge, ours is one of only two studies that have attempted to quantify individual additive intake, outside of population studies. Chazelas, Druesne-Pecollo [55] assessed data from the NutriNet-Santè cohort to qualify presence/absence of additives in brand foods using 3-large scale databases and report on the proportion of individuals who consumed common additives. Chazelas, Druesne-Pecollo [55] also estimated additive intake using both laboratory assays and based on Maximal Permissible Level of additives in the food published by the European Food Safety Authority (EFSA) and Joint FAO/WHO Expert Committee on Food Additives (JEFCA). In contrast, as discussed, we used validated questionnaires (12-month data) and 3-day food records to collect information on additives consumed, and estimated additive intakes based on Maximal Permissible Levels reported by JEFCA.

Our 12-month estimates were comparable to what has previously been reported in population level studies (Table 4) and in data from the NutriNet-Santè cohort. All additives we have reported on, other than polysorbate-80, titanium dioxide and aluminosilicateswere amongst the most commonly consumed additives in the NutriNet-Santè Cohort. Our estimates for consumed current (3 day) additive intake were lower than expected. Despite reports that food additives are ubiquitous in processed foods, we found that many did not contain any food additives. For example, in Australia, of the 1044 unique packaged foods consumed, 353 contained no additives.

Our estimated additive intakes fall within “acceptable” daily intake (ADI) levels (Table 4); however ADIs are based on toxicology and carcinogenesis, rather than the potential impact on the gut microbiome. The degree of intake that has clinical importance is yet to be determined.

### 4.3. Dietary Inflammatory Index

A recent approach to the inflammatory potential of food has been the development of quantitative “inflammatory indices”. The dietary inflammatory index (DII) used in this study was higher in Hong Kong than in Australia when all participants were combined and higher in Hong Kong Crohn’s disease patients than matched controls. The lack of difference in Australia between Crohn’s disease patients and control subjects may relate to the relatively high overall DII for all Australians. The association between the development of IBD and DII, and other index-based dietary patterns [66], has been investigated in several studies, but to our knowledge, this is the first to compare the DII of current diets of individuals with and without Crohn’s disease. The DII is derived from nutrient intake, with fibre and unsaturated fats considered anti-inflammatory and energy, total fat, and protein considered pro-inflammatory. There are mixed findings in relation to differences in intakes of these nutrients between individuals with and without Crohn’s disease [67,68]. While ultra-processed foods likely increase the overall inflammatory capacity of the diet, there are other ways that the DII score can be increased. To assess whether there was an interaction between overall inflammatory capacity and the quantity of additives in the diet, we assessed the correlation between total additive intake (mg/per kg body weight) and energy adjusted DII score. We found an association between the DII and current total additive intake in the cohort as a whole and within Crohn’s disease patients. Energy is one dietary component that contributes to an increased DII, however, in our study, dietary energy intake was not statically significantly different between Crohn’s disease patients and controls (Table 3, Supplementary Table S6). This finding suggests that it is inaccurate to assume that more energy-dense diets are always higher in total additive content and confirms that it is not necessary to correct total additive estimated based on energy intake.

There was a weak positive correlation between the DII and CRP in patients, this is in line with literature used to derive the DII [15] but in contrast to a study in Canadian patients with inactive Crohn’s disease, which found no association between DII and CRP [69]. There was no association between the DII and CDAI. Previous reports have found both no association [70], and a positive association between DII and the CDAI [71]. Use of varied methods to assess dietary intake and differences in cohorts may explain disparities. In particular, 85% of our cohort were in remission, which may have influenced our ability to detect an effect of diet on inflammation.

The data analysis was undertaken on the basis that each variable was an independent event [72]. The early life questionnaires assessed intake in age groups, thereby avoiding the need for age adjustment. 

### 4.4. Study Novelty and Strengths

Several studies have assessed pre-disease nutrient intake [22], dietary pattern [10], or dietary inflammatory potential [14], and the subsequent risk of developing Crohn’s disease. More recently, the association between pre-disease ultra-processed food intake and risk of developing IBD has been assessed in a respective review of data from the Nurses’ Health Study and Health Professionals follow-up study [50], and the abovementioned PURE cohort [36] and NutriNet-Santè cohort studies [55]. The PURE cohort was set up to assess associations between social, behavioral, genetic, and environmental factors and cardiovascular disease in low, middle and high-income countries. Diet was assessed using food frequency questionnaires (FFQs), which were validated to assess nutrient intake, with methodologies for assessing ultra-processed food intake being developed retrospectively. The NutriNet-Santè study is a web-based prospective cohort study that was established to investigate nutrition and health relationships; diet was assessed using repeated 24-h recalls, from which ultra-processed foods were quantified. Data from the NutriNet-Santè cohort was also used to quantify individual additive intake, however this was for the whole study population and no comparisons on the basis of health and disease, or geographical region, were made.

The ENIGMA study is comparable to the PURE cohort in its trans-national/ethnic design. The ENIGMA study is novel in that in contrast to the abovementioned studies, we utilised purpose-designed, IBD-specific, validated questionnaires to assess early life (0–18 years) processed food intake and quantify recent (last 12-month) dietary additive content of diets. Further, we used novel methodologies to estimate current additive intake based on 3-day food records. To our knowledge, this study is the first to evaluate Crohn’s disease patients’ and controls’ diet throughout life, using validated questionnaires. This is the first study to quantitate additive intake in individuals with Crohn’s disease based on specific brands of foods consumed, in contrast to previous studies, which focused only on ultra-processed food. To our knowledge, it is also the first to do this across regions of different economic development, culture, dietary patterns, and Crohn’s disease incidence.

### 4.5. Limitations

Study limitations included a reliance on retrospective recall of early life intake. In order to allow for the best possible recall, and in line with recommendations for questionnaire data used in epidemiological studies [73], our protocol allowed for participants to complete questionnaires over a week and check responses with parents. Questionnaires where then evaluated for completeness at research visits. Since the same process was applied to both Crohn’s disease patients and controls, any biases are likely to be systematic and impact all participants equally. However, recall bias in patients with a disease that focuses them on food cannot be excluded. During pilot testing, recall was assessed twice two-weeks apart and moderate to excellent reliability was demonstrated, confirming that participants’ estimates of early life dietary intake was consistent over time. Individuals (and their parents or guardians) recall of diet intake in the past has been assessed in several longitudinal studies. The correlation between prospectively recorded intake and retrospectively recalled intake over the past 10 to 40 years is comparable to the correlation between prospectively recalled intake and retrospectively recalled intake recalled from the preceding 6 to 12 months [33]. This indicates that recall bias in our study may be similar to bias in studies assessing only recent intake.

Estimations of food additive intake were based on the estimated maximum, rather than actual, presence of additives in foods, and recent (12-month) data was not brand specific. Questions related to processed vegetables may not adequately distinguish between fermented vegetables without additives and non-fermented processed vegetables. In addition, we focused on select emulsifiers, artificial sweeteners, titanium dioxide (a food colorant) and aluminosilicates (anti-caking agents). Other additives may require future attention. These include the colorants Red 40 and Yellow 6, which recently have been shown to induce colitis in mice-models with dysregulated expression of interleukin 23 [74], as well as other emulsifiers, such sunflower lecithin, guar gum and agar, which have been shown to have negative compositional and functional effects on the human microbiome ex vivo, and soy lecithin and mono/di-glycerides of fatty acids, which do not appear to impact the human gut microbiome [75].

## 5. Conclusions

In conclusion, early life intake of food additives is more common in Crohn’s disease patients than a range of control subjects, across different geographic and ethnic regions. These data add weight to the hypothesis that such additives are a permissive environmental factor that facilitates the emergence of Crohn’s disease in susceptible individuals. Current food additive intake is also more common in patients with Crohn’s disease, potentially contributing to the presence of ongoing inflammation. These findings have implication in disease prevention, and as an aid to the treatment of existing disease.

## Figures and Tables

**Table 1:** Patient Characteristics.

		All Regions Combined (n = 531)				Australia (n = 180)				Hong Kong (n = 160)				Mainland China (n = 191)			
	**All Subjects Combined**	CD (274)	HHM (83)	FDR (82)	HC (92)	CD (100)	HHM (32)	FDR (28)	HC (20)	CD (95)	HHM (22)	FDR (21)	HC (22)	CD (79)	HHM (29)	FDR (33)	HC (50)
**Gender** **Male**	297 (53)	160 (59)	55 (47)	39 (48)	43 (47)	49 (49)	25 (50)	9 (32)	8 (40)	64 (68)	15 (39)	7 (33)	9 (41)	47 (60)	15 (52)	23 (70)	26 (52)
n (%)																	
**Age**	43	36	47.5	49	47	38.1	46.8	50.2	51.1	39.7	50.9	55.4	47.9	36	38	43	42
(median, year)																	
**BMI**	23.1	22.5	23.8	24.2	22.6	25.1	27.6	25.9	27.4	22.7	23.9	25.5	23.7	19.6	22.3	21.5	22
(median, kg/m^2^)																	

CD—Crohn’s disease cases, HHM—Healthy household members, FDR—First-degree relatives, HC—Healthy unrelated controls, BMI - Body Mass Index.

**Table 2:** Disease diagnosis and disease activity of Australia and Hong Kong Crohn’s disease participants.

	All	Australia	Hong Kong	China	*p*-Value
**Age at Diagnosis**	25.0	22.0	27.5	37.9	
(median, years [IQR, Range])	(18, 7–69)	(13, 7–65)	(22, 8–69)	(24, 19–68)	0.029
**Time since Diagnosis**	8.0	9.0	6.0	3.0	
(median, years ([IQR, Range])	(13, 0–41)	(12, 0–41)	(12, 0–30)	(4, 1–23)	0.048
**CDAI**					
median (IQR)	63.9 (78.4)	48.6 (73.4)	74.3 (75.2)		<0.001
Remission (%)	85.3	85.7	84.9		0.910
Mildly active (%)	12.0	11.2	12.8		
Moderately active (%)	2.7	3.1	2.3		
**CRP**					
median (IQR)	0.9 (5.1)	0.0 (4.0)	2.6 (6.2)	4.1 (6.1)	<0.001
Normal (%)	70.4	74.2	66.7	69.7	0.261
Above normal (%)	29.6	25.8	33.3	30.3	
**Hemoglobin**					
(median, IQR))	138.0 (18.0)	135.5 (20.55)	136.0 (20.0)	142.4 (2.65)	0.066
Normal (%)	77.1	80.6	73.7	72.0	0.256
Below normal (%)	22.9	19.4	26.3	28.0	

**Table 3:** Summary of significant differences in usual recent 12-month food additive intake across regions and between Crohn’s disease participants and various controls.

Dietary Inflammatory Index (DII)	All participants across all regions: ↑ HK vs. AUS
Food Additives	
Total Additive Intake	**Recent (12 months)** All Crohn’s Cases: ↑HK vs. MC; ↑AUS vs. MC ↑ Crohn’s Cases vs. Controls (ALL, AUS) ↑ Crohn’s Cases vs. Healthy Household Members (HK) ↑ Crohn’s Cases vs. Healthy Unrelated Controls (ALL) **Current (last 3 days)** ↑ Crohn’s Cases vs. Healthy Household Members (AUS, HK) ↑ Crohn’s Cases vs. First Degree Relatives (AUS + HK combined)
Total Artificial Sweetener Intake	**Recent (12 months)** All participants across all regions: ↑AUS vs. MC; ↑HK vs. MC All Crohn’s Cases: ↑AUS vs. MC ↑ Crohn’s Cases vs. Controls (ALL, HK) ↑ Crohn’s Cases vs. Healthy Household Members (ALL, HK) ↑ Crohn’s Cases vs. First-degree Relatives (ALL, HK) ↑ Crohn’s Cases vs. Healthy Unrelated Controls (ALL)
Total Emulsifier Intake	**Recent (12 months)** All participants across all regions: ↑AUS vs. MC; ↑HK vs. MC Crohn’s Cases: ↑AUS vs. MC; ↑HK vs. MC ↑ Crohn’s Cases vs. Controls (ALL, MC) ↑ Crohn’s Cases vs. Healthy Household Members (ALL, MC) ↑ Crohn’s Cases vs. First-degree Relatives (ALL, AUS) ↑ Crohn’s Cases vs. Healthy Unrelated Controls (ALL) **Current (last 3 days):** ↑ Crohn’s Cases vs. Controls (ALL, MC) ↑ Crohn’s Cases vs. Healthy Household Members (HK)
Energy (calories)	**Current (last 3 days)** All participants across all regions: ↑AUS vs. HK
Individual Additives	
Polysorbate-80	**Recent (12-month)** ↑ Crohn’s Cases vs. Controls (ALL) ↑ Crohn’s Cases vs. Healthy Household Members: ↑ALL: CD; ↑AUS: CD; ↑MC: CD ↑ Crohn’s Cases vs. First-degree Relatives (ALL, AUS, HK) ↑ Crohn’s Cases vs. Healthy Unrelated Controls (ALL, AUS)
Carboxymethylcellulose	**Recent (12-month)** ↑ Crohn’s Cases vs. Controls (AUS) ↑ Crohn’s Cases vs. Healthy Household Members (MC) ↑ Crohn’s Cases vs. First-degree Relatives (ALL, HK) ↑ Crohn’s Cases vs. Healthy Unrelated Controls (ALL) **Current (last 3 days)** ↑ Crohn’s Cases vs. Healthy Household Members (AUS + HK)
Carrageenan	**Recent (12-month)** All subjects: ↑AUS vs. HK; ↑AUS vs. MC; ↑HK vs. MC All Crohn’s cases: ↑AUS vs. HK; ↑AUS vs. MC; ↑HK vs. MC ↑ Crohn’s Cases vs. Controls (ALL, MC) ↑ Crohn’s Cases vs. Healthy Household Members (MC) ↑ Crohn’s Cases vs. First-degree Relatives (ALL) ↑ Crohn’s Cases vs. Healthy Unrelated Controls (ALL, MC)
Aluminosilicates	**Recent (12-month)** ↑ Crohn’s Cases vs. Controls (ALL) ↑ Crohn’s Cases vs. First-degree Relatives (ALL, HK, MC) ↑ Crohn’s Cases vs. Healthy Unrelated Controls (ALL, MC)
Sulphites	**Recent (12 months)** All participants across all regions: ↑AUS vs. HK; ↑AUS vs. MC; ↑HK vs. MC Crohn’s Cases: ↑AUS vs. HK; ↑AUS vs. MC; ↑HK vs. MC ↑ Crohn’s Cases vs. Controls (ALL) ↓ Crohn’s Cases vs. First-degree Relatives (MC) ↑ Crohn’s Cases vs. Healthy Unrelated Controls (ALL) **Current (last 3 days):** All participants across all regions: ↑AUS vs. HK ↑ Crohn’s Cases vs. Healthy Unrelated Controls (AUS)
Titanium Dioxide	**Recent (12-month)** ↑ Crohn’s Cases vs. Controls (ALL) ↑ Crohn’s Cases vs. Healthy Household Members: ↑CD (ALL) ↑ Crohn’s Cases vs. First-degree Relatives (ALL, HK) ↑ Crohn’s Cases vs. Healthy Unrelated Controls (ALL, HK) ↓ Crohn’s Cases vs. Healthy Unrelated Controls (MC)
Aspartame	**Recent (12-month)** All participants across all regions: ↑AUS vs. HK; ↑AUS vs. MC; ↑HK vs. MC All Crohn’s Cases: ↑AUS vs. HK; ↑AUS vs. MC; ↑HK vs. MC ↑ Crohn’s Cases Controls (ALL, MC) ↑ Crohn’s Cases vs. Healthy Household Members (HK) ↑ Crohn’s Cases vs. First-degree Relatives (HK) ↑ Crohn’s Cases vs. Healthy Unrelated Controls **Current (last 3 days):** All participants across all regions: ↑AUS vs. HK All Crohn’s cases: ↑AUS vs. HK
Sucralose	**Recent (12-month)** ↑ Crohn’s Cases vs. Controls (ALL) ↑ Crohn’s Cases vs. Healthy Household Members (ALL) ↑ Crohn’s Cases vs. First-degree Relatives (ALL, HK) ↑ Crohn’s Cases vs. Healthy Unrelated Controls (ALL)
Saccharin	**Recent (12-month)** ↑ Crohn’s Cases vs. Controls (ALL) ↑ Crohn’s Cases vs. First-degree Relatives (AUS, HK) ↑ Crohn’s Cases vs. Healthy Unrelated Controls (ALL)

Significant at *p* < 0.05; ALL—All regions combined, AUS—Australia, HK—Hong Kong, MC—Mainland China. ↑ Increased intake versus comparator, ↓ decreased intake versus comparator. Mainland China participants did not complete current intake data.

**Table 4:** Estimated usual and current (3 day) intake of additives in mg/kg body weight/day and comparisons to acceptable daily intake and estimated population intakes in various countries.

Additive	Usual Intake	Recent Intake (Mean or Median)	Acceptable Daily Intake (ADI) mg/kg Body Weight/Day	Population	Estimated Population Intakes (Tier Estimate)
Polysorbate-80	11.3	0.00 (mean) 0.09 (median)	0–25	USA, 2 years and older	5–10 (T2) [56]
				USA, 2 years and older	5 (T1) [56]
				Europe (UK, Ireland, France, Italy), 8 years to 97 years	1.850–2.218 (T2) [57] 0.610–1.177 (T3) [57] includes all polysorbates
Carboxymethylcellulose	19.5	0.18 (mean)	Not specified	USA, 2 years and older	24–30 (T2) [56]
		0.00 (median)			
Carrageenan	13.9	0.17 (mean)	Not specified	Finland, whole population	0.67 * [58] 18.07 (female)–31.19 (male) (T3) ** [59] 18.96 [55]
		0.00 (median)		USA (Florida), over 17 years France, 18 years and older	
Aluminosilicates	0.04	0	Not specified	N/A	Not available
Sulphites	0.2	0.00 (median)	0–0.7	Europe (UK, Ireland, France, Italy), 8 years to 97 years New Zealand, 15 years and olderFinland, whole population France, 18 years and older	0.296–0.620 (Europe, T2) [57] 0.017–0.132 (Europe, T3) [57] 0.05 (>65 years female) 0.27 (19–24 years female) (New Zealand, T3) [60] 0.05 * [58] 0.00 [55]
		0.03 (mean)			

Titanium Dioxide	0.2	0.11 (mean) (median)	Not limited	Dutch, 7 to 69 years	0.17 (T3 ***) [61]
Aspartame	1.9	0.0 (median)	0–40	Europe (UK, Ireland, France, Italy), 8 years to 97 years	0.957–4.928 (Europe, T2) [57] 0.182–1.018 (Europe, T3) [57]
				Belgium, 15 years and older	1.95 (T2) [62]
		0.19 (mean)		Belgium, 15 years and older	0.60 (T3) [62]
				Japan, 20 years and older	0.0435 (adults) [63] 0.0320 (elderly)(T3) [63]
				Ireland, 18 to 90 years France, 18 years and older	1.05 (T1) [64] 0.62 (T2) [64] 0.28 (T3) [64] 0.000 [55]
Sucralose	0.8	0 (median)	0–15	Korea, whole population	0.059 (T3); 0.044 (>65 years) to 0.95 (1–2 years) [65]
		0.006 (mean)		Belgium, 15 years and older	0.42 [62]
				Ireland, 18 to 90 years France, 18 years and older	0.06 (T1) [64] 0.09 (T2) [64] 0.05(T3) [64] 0.000 [55]
Saccharin	0.6	0	0–5	Korea, whole population Ireland, 18 to 90 years Finland, whole population France, 18 years and older	0.0832 (T3); 0.049 (>65 years) to 0.1 (20–29 years) [65]
					0.34 (T2) [62] 0.15 (T3) [62] 0.24 (T1) [64] 0.06 (T2) [64] 0.03 (T3) [64]
					0.8 * [58] 0.00 [55]

**T1 = Tier 1 Estimates:** Theoretical food consumption data and Maximal Permissible Level for the additive [54]; **T2 = Tier 2 Estimates:** Actual national food consumption data and Maximal Permissible Level for the additive [54]; **T3 = Tier 3 Estimates:** Actual national food consumption data and Maximal Permissible Level for the additive and actual use levels of the additive in foods [54] * Converted from g/person to mg/kg body weight based on average weight in population of 74.5 kg. Theoretical food consumption data (food balance sheets, food production tables) and actual use level of the additives in foods (analytical studies) ** Actual food consumption data (purpose-designed questionnaire) and actual use levels (industry data) *** Analytical studies performed on some individual foods and extrapolated to similar foods.

## Data Availability

Data described in the manuscript, code book, and analytic code will be made available upon request pending approval.

## Supplementary Materials

The following supporting information can be downloaded at: https://www.mdpi.com/article/10.3390/nu14173627/s1, Figure S1: Participant Flowchart; Table S1: Summary of Group Comparisons in The ENIGMA Study; Table S2: Definitions and Examples of Processed Foods; Table S3: Proportion of Individuals who consumed processed foods on a weekly basis in Early Life; Table S4: Median annual intake of additives calculated based on responses to the survey on intake of processed foods consumed in the past 12 months; Table S5: Median recent intake of additives calculated based on maximal permitted level of additives present in foods recorded in 3-day food diaries; Table S6: Median Recent Intake of Energy, Nutrients and Alcohol, Estimated Based on Foods Recorded in 3-Day Food Diaries; Table S7: Association Between DII, Additives, Nutrients (based on 3-day food diaries), and Crohn’s disease Activity Index (CDAI), C-Reactive Protein (CRP) and Hemoglobin (Hb) in Australia and Hong Kong Crohn’s disease Patients; Table S8: Correlation between energy-adjusted DII and current, total additive intake (mg/per kg body weight); Table S9: Differences in Current (3 day) Intake of Energy, Nutrients, and Additives Between Crohn’s disease Participants With Normal Versus Elevated CRP, and Normal Versus Low Hemoglobin (Hb)

## References

[1] Liu J.Z., Van Sommeren S., Huang H., Ng S.C., Alberts R., Takahashi A., Ripke S., Lee J.C., Jostins L., Shah T. (2015). Association analyses identify 38 susceptibility loci for inflammatory bowel disease and highlight shared genetic risk across populations. Nat. Genet..

[2] Ko Y., Kariyawasam V., Karnib M., Butcher R., Samuel D., Alrubaie A., Rahme N., McDonald C., Cowlishaw J., Katelaris P. (2015). Inflammatory Bowel Disease Environmental Risk Factors: A Population-Based Case–Control Study of Middle Eastern Migration to Australia. Clin. Gastroenterol. Hepatol..

[3] Ng S.C., Bernstein C.N., Vatn M.H., Lakatos P.L., Loftus E.V., Tysk C., O’Morain C., Moum B., Colombel J.-F., on behalf of the Epidemiology and Natural History Task Force of the International Organization of Inflammatory Bowel Disease (IOIBD) (2013). Geographical variability and environmental risk factors in inflammatory bowel disease. Gut.

[4] Ng S.C., Tang W., Leong R.W., Chen M., Ko Y., Studd C., Niewiadomski O., Bell S., Kamm M.A., De Silva H.J. (2015). Environmental risk factors in inflammatory bowel disease: A population-based case-control study in Asia-Pacific. Gut.

[5] Ng S.C., Shi H.Y., Hamidi N., Underwood F.E., Tang W., Benchimol E.I., Panaccione R., Ghosh S., Wu J.C.Y., Chan F.K.L. (2017). Worldwide incidence and prevalence of inflammatory bowel disease in the 21st century: A systematic review of population-based studies. Lancet.

[6] Ahuja V., Tandon R.K. (2010). Inflammatory bowel disease in the Asia-Pacific area: A comparison with developed countries and regional differences. J. Dig. Dis..

[7] Lewis J.D., Abreu M.T. (2017). Diet as a Trigger or Therapy for Inflammatory Bowel Diseases. Gastroenterology.

[8] Kaplan G.G., Ng S.C. (2017). Understanding and Preventing the Global Increase of Inflammatory Bowel Disease. Gastroenterology.

[9] Levine A., Boneh R.S., Wine E. (2018). Evolving role of diet in the pathogenesis and treatment of inflammatory bowel diseases. Gut.

[10] Li T., Qiu Y., Yang H.S., Li M.Y., Zhuang X.J., Zhang S.H., Feng R., Chen B.L., He Y., Zeng Z.R. (2020). Systematic review and meta-analysis: Association of a pre-illness Western dietary pattern with the risk of developing inflammatory bowel disease. J. Dig. Dis..

[11] Khorshidi M., Djafarian K., Aghayei E., Shab-Bidar S. (2020). A posteriori dietary patterns and risk of inflammatory bowel disease: A meta-analysis of observational studies. Int. J. Vitam. Nutr. Res..

[12] Vasseur P., Dugelay E., Benamouzig R., Savoye G., Lan A., Srour B., Hercberg S., Touvier M., Hugot J.-P., Julia C. (2021). Dietary Patterns, Ultra-processed Food, and the Risk of Inflammatory Bowel Diseases in the NutriNet-Santé Cohort. Inflamm. Bowel Dis..

[13] Khalili H., Håkansson N., Chan S.S., Chen Y., Lochhead P., Ludvigsson J., Chan A.T., Hart A.R., Olén O., Wolk A. (2020). Adherence to a Mediterranean diet is associated with a lower risk of later-onset Crohn’s disease: Results from two large prospective cohort studies. Gut.

[14] Lo C.-H., Lochhead P., Khalili H., Song M., Tabung F.K., Burke K.E., Richter J.M., Giovannucci E.L., Chan A.T., Ananthakrishnan A.N. (2020). Dietary Inflammatory Potential and Risk of Crohn’s Disease and Ulcerative Colitis. Gastroenterology.

[15] Shivappa N., Steck S.E., Hurley T.G., Hussey J.R., Hebert J.R. (2014). Designing and developing a literature-derived population-based dietary inflammatory index. Public Health Nutr..

[16] Shivappa N., Hebert J.R., Rashvand S., Rashidkhani B., Hekmatdoost A. (2016). Inflammatory potential of diet and risk of ulcerative colitis in a case-control study from Iran. Nutr. Cancer.

[17] Marx W., Veronese N., Kelly J.T., Smith L., Hockey M., Collins S., Trakman G.L., Hoare E., Teasdale S.B., Wade A. (2021). The Dietary Inflammatory Index and Human Health: An Umbrella Review of Meta-Analyses of Observational Studies. Adv. Nutr. Int. Rev. J..

[18] Yuan F., Deng L., Sun X., Chen Z., Shivappa N., Sheth A.K., Cooper G.S., Hebert J.R., Li L. (2021). Dietary inflammatory index and risk of colorectal adenoma: Effect measure modification by race, nonsteroidal anti-inflammatory drugs, cigarette smoking and body mass index?. Cancer Causes Control.

[19] da Silva A., Felício M.B., Caldas A.P.S., Miranda Hermsdorff H.H., Bersch-Ferreira Â C., Torreglosa C.R., Shivappa N., Hébert J.R., Weber B., Bressan J. (2020). Pro-inflammatory diet is associated with a high number of cardiovascular events and ultra-processed foods consumption in patients in secondary care. Public Health Nutr..

[20] Piovani D., Danese S., Peyrin-Biroulet L., Nikolopoulos G.K., Lytras T., Bonovas S. (2019). Environmental Risk Factors for Inflammatory Bowel Diseases: An Umbrella Review of Meta-analyses. Gastroenterology.

[21] Spooren C.E.G.M., Pierik M.J., Zeegers M.P., Feskens E.J.M., Masclee A.A.M., Jonkers D.M.A.E. (2013). Review article: The association of diet with onset and relapse in patients with inflammatory bowel disease. Aliment. Pharmacol. Ther..

[22] Hou J.K., Abraham B., El-Serag H. (2011). Dietary Intake and Risk of Developing Inflammatory Bowel Disease: A Systematic Review of the Literature. Am. J. Gastroenterol..

[23] Chassaing B., Koren O., Goodrich J.K., Poole A.C., Srinivasan S., Ley R.E., Gewirtz A.T. (2015). Dietary emulsifiers impact the mouse gut microbiota promoting colitis and metabolic syndrome. Nature.

[24] Chassaing B., Van De Wiele T., De Bodt J., Marzorati M., Gewirtz A.T. (2017). Dietary emulsifiers directly alter human microbiota composition and gene expression ex vivo potentiating intestinal inflammation. Gut.

[25] Roberts C.L., Keita Å.V., Duncan S.H., O’Kennedy N., Söderholm J.D., Rhodes J.M., Campbell B.J. (2010). Translocation of Crohn’s disease *Escherichia coli* across M-cells: Contrasting effects of soluble plant fibres and emulsifiers. Gut.

[26] Ruiz P.A., Morón B., Becker H.M., Lang S., Atrott K., Spalinger M.R., Scharl M., Wojtal K.A., Fischbeck-Terhalle A., Frey-Wagner I. (2017). Titanium dioxide nanoparticles exacerbate DSS-induced colitis: Role of the NLRP3 inflammasome. Gut.

[27] Lomer M.C.E., Thompson R.P.H., Powell J.J. (2002). Fine and ultrafine particles of the diet: Influence on the mucosal immune response and association with Crohn’s disease. Proc. Nutr. Soc..

[28] Zhang J., Hoedt E.C., Liu Q., Berendsen E., Teh J.J., Hamilton A., Brien A.W.O., Ching J.Y., Wei H., Yang K. (2021). Elucidation of *Proteus mirabilis* as a Key Bacterium in Crohn’s Disease Inflammation. Gastroenterology.

[29] Pitcher M.C., Cummings J.H. (1996). Hydrogen sulphide: A bacterial toxin in ulcerative colitis?. Gut.

[30] Ronan V., Yeasin R., Claud E.C. (2021). Childhood Development and the Microbiome—The Intestinal Microbiota in Maintenance of Health and Development of Disease During Childhood Development. Gastroenterology.

[31] Zhao J., Ng S.C., Lei Y., Yi F., Li J., Yu L., Zou K., Dan Z., Dai M., Ding Y. (2013). First Prospective, Population-Based Inflammatory Bowel Disease Incidence Study in Mainland of China: The Emergence of “Western” Disease. Inflamm Bowel Dis..

[32] Ng S.C., Leung W.K., Shi H.Y., Li M.K.K., Leung C.M., Ng C.K.M., Lo F.H., Hui Y.T., Tsang S.W.C., Chan Y.K. (2016). Epidemiology of Inflammatory Bowel Disease from 1981 to 2014: Results from a Territory-Wide Population-Based Registry in Hong Kong. Inflamm. Bowel Dis..

[33] Trakman G.L., Lin W., Wilson-O’Brien A.L., Stanley A., Hamilton A.L., Tang W., Or L., Ching J., Morrison M., Yu J. (2020). Development and Validation of Surveys to Estimate Food Additive Intake. Nutrients.

[34] Marion-Letellier R., Amamou A., Savoye G., Ghosh S. (2019). Inflammatory bowel diseases and food additives: To add fuel on the flames!. Nutrients.

[35] Hviid A., Svanström H., Frisch M. (2011). Antibiotic use and inflammatory bowel diseases in childhood. Gut.

[36] Narula N., Wong E.C.L., Dehghan M., Mente A., Rangarajan S., Lanas F., Lopez-Jaramillo P., Rohatgi P., Lakshmi P.V.M., Varma R.P. (2021). Association of ultra-processed food intake with risk of inflammatory bowel disease: Prospective cohort study. BMJ.

[37] (2018). Cint Insight Exchange. “Australia: How Often Do You Eat Fast Food (Any Quick Service Restaurant) in Any Given Week (on Average)?”. *Statista*, Australia. https://www.statista.com/statistics/921343/australia-average-fast-food-consumption-per-week/.

[38] Prideaux L., Kang S., Wagner J., Buckley M., Mahar J.E., De Cruz P., Wen Z., Chen L., Xia B., van Langenberg D.R. (2013). Impact of Ethnicity, Geography, and Disease on the Microbiota in Health and Inflammatory Bowel Disease. Inflamm. Bowel Dis..

[39] Niewiadomski O., Studd C., Wilson J., Williams J., Hair C., Knight R., Prewett E., Dabkowski P., Alexander S., Allen B. (2016). Influence of food and lifestyle on the risk of developing inflammatory bowel disease. Intern. Med. J..

[40] Persson P.G., Ahlbom A., Hellers G. (1992). Diet and Inflammatory Bowel Disease: A Case-Control Study. Epidemiology.

[41] Jimenez Loayza J.J., Berendsen E.M., Teh J.J., Hoedt E.C., Zhang J., Liu Q., Hamilton A.L., Wilson-O’Brien A., Trakman G.L., Lin W. (2019). P837 The common food additives sodium sulfite and polysorbate 80 have a profound inhibitory effect on the commensal, anti-inflammatory bacterium *Faecalibacterium prausnitzii*: The ENIGMA study. J. Crohn’s Colitis.

[42] Wang Z., Shao Y. (2018). Effects of microbial diversity on nitrite concentration in pao cai, a naturally fermented cabbage product from China. Food Microbiol..

[43] Zhang G., Lu M., Liu R., Tian Y., Vu V.H., Li Y., Liu B., Kushmaro A., Li Y., Sun Q. (2020). Inhibition of *Streptococcus mutans* Biofilm Formation and Virulence by *Lactobacillus plantarum* K41 Isolated From Traditional Sichuan Pickles. Front. Microbiol..

[44] Zhang J., Chen B., Liu B., Zhou X., Mu J., Wang Q., Zhao X., Yang Z. (2018). Preventive Effect of *Lactobacillus fermentum* CQPC03 on Activated Carbon-Induced Constipation in ICR Mice. Medicina.

[45] Dimidi E., Cox S.R., Rossi M., Whelan K. (2019). Fermented Foods: Definitions and Characteristics, Impact on the Gut Microbiota and Effects on Gastrointestinal Health and Disease. Nutrients.

[46] Campbell E., Nagler C.R. (2021). Fe, fi, fo, fum, I smell the diet of a healthy human. Cell.

[47] Wastyk H.C., Fragiadakis G.K., Perelman D., Dahan D., Merrill B.D., Yu F.B., Topf M., Gonzalez C.G., Van Treuren W., Han S. (2021). Gut-microbiota-targeted diets modulate human immune status. Cell.

[48] I Benchimol E., Mack D.R., Guttmann A., Nguyen G.C., To T., Mojaverian N., Quach P., Manuel D.G. (2015). Inflammatory Bowel Disease in Immigrants to Canada And Their Children: A Population-Based Cohort Study. Am. J. Gastroenterol..

[49] Agrawal M., Corn G., Shrestha S., Nielsen N.M., Frisch M., Colombel J.-F., Jess T. (2020). Inflammatory bowel diseases among first-generation and second-generation immigrants in Denmark: A population-based cohort study. Gut.

[50] Lo C.-H., Khandpur N., Rossato S.L., Lochhead P., Lopes E.W., Burke K.E., Richter J.M., Song M., Korat A.V.A., Sun Q. (2021). Ultra-processed Foods and Risk of Crohn’s Disease and Ulcerative Colitis: A Prospective Cohort Study. Clin. Gastroenterol. Hepatol..

[51] Swidsinski A., Ung V., Sydora B.C., Loening-Baucke V., Doerffel Y., Verstraelen H., Fedorak R.N. (2009). Bacterial Overgrowth and Inflammation of Small Intestine After Carboxymethylcellulose Ingestion in Genetically Susceptible Mice. Inflamm. Bowel Dis..

[52] Devkota S., Chang E.B. (2015). Interactions between diet, bile acid metabolism, gut microbiota, and inflammatory bowel diseases. Dig. Dis..

[53] Ijssennagger N., van der Meer R., van Mil S.W. (2016). Sulfide as a mucus barrier-breaker in inflammatory bowel disease?. Trends Mol. Med..

[54] Cox S., Sandall A., Smith L., Rossi M., Whelan K. (2020). Food additive emulsifiers: A review of their role in foods, legislation and classifications, presence in food supply, dietary exposure, and safety assessment. Nutr. Rev..

[55] Chazelas E., Druesne-Pecollo N., Esseddik Y., de Edelenyi F.S., Agaesse C., De Sa A., Lutchia R., Rebouillat P., Srour B., Debras C. (2021). Exposure to food additive mixtures in 106,000 French adults from the NutriNet-Santé cohort. Sci. Rep..

[56] Shah R., Kolanos R., DiNovi M.J., Mattia A., Kaneko K.J. (2017). Dietary exposures for the safety assessment of seven emulsifiers commonly added to foods in the United States and implications for safety. Food Addit. Contam. Part A.

[57] Vin K., Connolly A., McCaffrey T., McKevitt A., O’Mahony C., Prieto M.A., Tennant D., Hearty A., Volatier J.L. (2013). Estimation of the dietary intake of 13 priority additives in France, Italy, the UK and Ireland as part of the FACET project. Food Addit. Contam. Part A.

[58] Penttila P.L., Salminen S., Niemi E. (1988). Estimates on the intake of food additives in Finland. Z Lebensm Unters Forsch..

[59] Shah Z.C., Huffman F.G. (2003). Current Availability and Consumption of Carrageenan-Containing Foods. Ecol. Food Nutr..

[60] Cressey P., Jones S. (2009). Levels of preservatives (sulfite, sorbate and benzoate) in New Zealand foods and estimated dietary exposure. Food Addit. Contam. Part A.

[61] Rompelberg C., Heringa M.B., Van Donkersgoed G., Drijvers J., Roos A., Westenbrink S., Peters R., Van Bemmel G., Brand W., Oomen A.G. (2016). Oral intake of added titanium dioxide and its nanofraction from food products, food supplements and toothpaste by the Dutch population. Nanotoxicology.

[62] Huvaere K., Vandevijvere S., Hasni M., Vinkx C., Van Loco J. (2012). Dietary intake of artificial sweeteners by the Belgian population. Food Addit. Contam. Part A.

[63] Ishiwata H., Yamada T., Yoshiike N., Nishijima M., Kawamoto A., Uyama Y. (2002). Daily intake of food additives in Japan in five age groups estimated by the market basket method. Eur. Food Res. Technol..

[64] Buffini M., Goscinny S., Van Loco J., Nugent A.P., Walton J., Flynn A., Gibney M.J., McNulty B.A. (2018). Dietary intakes of six intense sweeteners by Irish adults. Food Addit Contam. Part A Chem. Anal. Control. Exp. Risk Assess.

[65] Suh H.-J., Choi S. (2013). Use of Sodium Saccharin and Sucralose in Foodstuffs and the Estimated Daily Intakes of Both Products in Korea. Korean J. Food Sci. Technol..

[66] Tian Z., Zhuang X., Zhao M., Zhuo S., Li X., Ma R., Li N., Liu C., Zhu Y., Tang C. (2021). Index-Based Dietary Patterns and Inflammatory Bowel Disease: A Systematic Review of Observational Studies. Adv. Nutr. Int. Rev. J..

[67] Principi M., Losurdo G., Iannone A., Contaldo A., Deflorio V., Ranaldo N., Pisani A., Ierardi E., Di Leo A., Barone M. (2018). Differences in dietary habits between patients with inflammatory bowel disease in clinical remission and a healthy population. Ann Gastroenterol.

[68] Opstelten J.L., de Vries J.H., Wools A., Siersema P.D., Oldenburg B., Witteman B.J. (2018). Dietary intake of patients with inflammatory bowel disease: A comparison with individuals from a general population and associations with relapse. Clin. Nutr..

[69] Naqvi S.A., Taylor L.M., Panaccione R., Ghosh S., Barkema H.W., Hotte N., Shommu N., Kaur S., Reimer R.A., Madsen K.L. (2021). Dietary patterns, food groups and nutrients in Crohn’s disease: Associations with gut and systemic inflammation. Sci. Rep..

[70] Mirmiran P., Moslehi N., Morshedzadeh N., Shivappa N., Hébert J.R., Farsi F., Daryani N.E. (2019). Does the inflammatory potential of diet affect disease activity in patients with inflammatory bowel disease?. Nutr. J..

[71] Lamers C.R., De Roos N.M., Witteman B.J.M. (2020). The association between inflammatory potential of diet and disease activity: Results from a cross-sectional study in patients with inflammatory bowel disease. BMC Gastroenterol..

[72] Rothman K.J. (1990). No adjustments are needed for multiple comparisons. Epidemiology.

[73] Friedenreich C. (1994). Improving long-term recall in epidemiologic studies. Epidemiology.

[74] He Z., Chen L., Catalan-Dibene J., Bongers G., Faith J.J., Suebsuwong C., DeVita R.J., Shen Z., Fox J.G., Lafaille J.J. (2021). Food colorants metabolized by commensal bacteria promote colitis in mice with dysregulated expression of interleukin-23. Cell Metab..

[75] Naimi S., Viennois E., Gewirtz A.T., Chassaing B. (2021). Direct impact of commonly used dietary emulsifiers on human gut microbiota. Microbiome.

