# Supplementary material for: Processed Food as a Risk Factor for the Development and Perpetuation of Crohn’s Disease—The ENIGMA Study
Source: Nutrients. 2022 Sep 2;14(17):3627. doi: 10.3390/nu14173627 (PMC9460819; doi:10.3390/nu14173627)
Supplement: Supplementary file 1 [file nutrients-14-03627-s001.zip › nutrients-1859149-supplementary.pdf]

Food as a Risk Factor for the Development and Perpetuation of Crohn's Disease.
The ENIGMA Study

Supplementary Materials

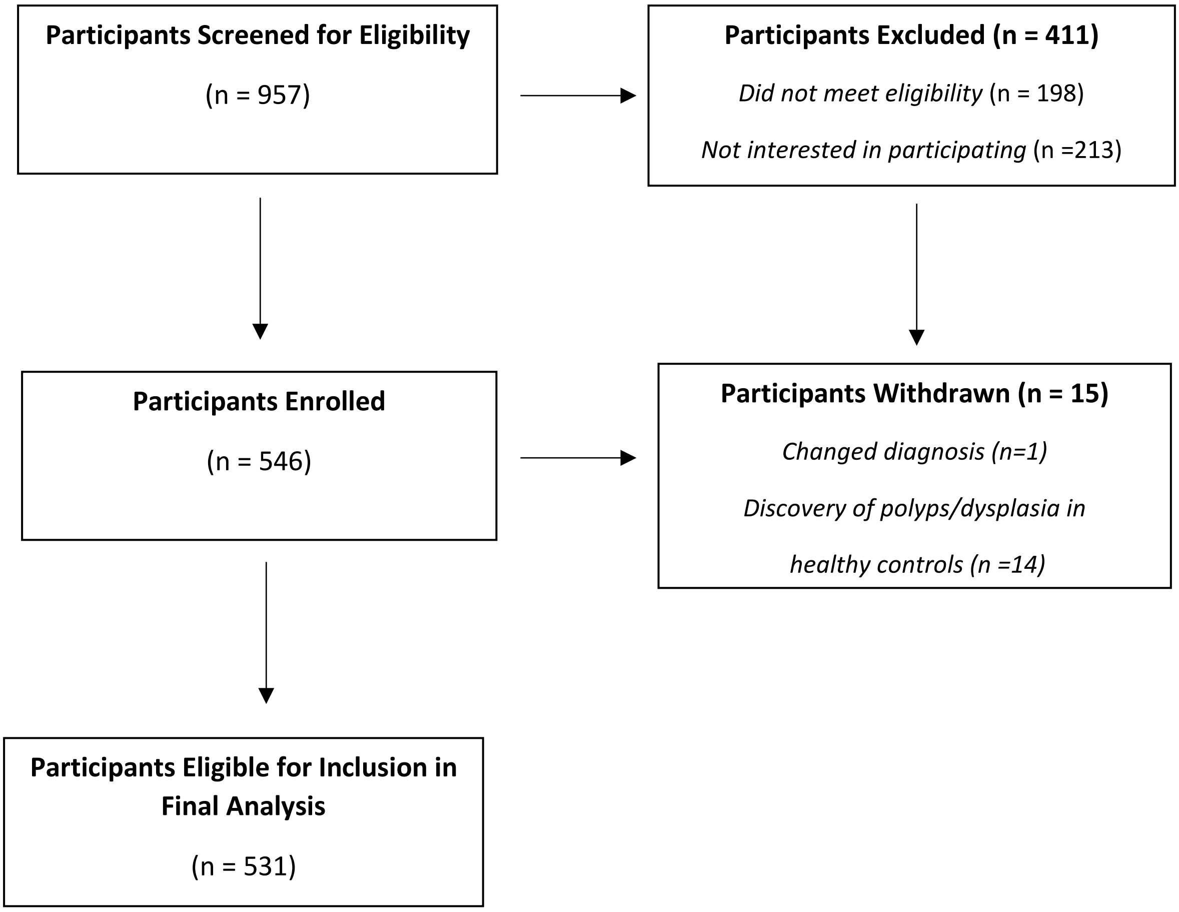

Supplementary Figure S1: Participant Flowchart

Supplementary Table S1. Summary of Group Comparisons in The ENIGMA Study

**Regional comparisons**		
All Australia	VS	All Hong Kong
All Australia	VS	All Mainland China
All Hong Kong	VS	All Mainland China
**Regional Crohn's disease comparisons**		
Australia Crohn's disease cases	VS	Hong Kong Crohn's disease cases
Australia Crohn's disease cases	VS	Hong Kong Crohn's disease cases
Hong Kong Crohn's disease cases	VS	Mainland China Crohn's disease cases
**Participant type comparisons**		
Australia Crohn's disease cases	VS	Australia non-Crohn's disease controls (all controls)<br>Australia first degree relatives (paired and unpaired)<br>Australia healthy household members (paired and unpaired)<br>Australia healthy unrelated controls
Hong Kong Crohn's disease cases	VS	Hong Kong non-Crohn's disease controls (all controls)<br>Hong Kong first degree relatives (paired and unpaired)<br>Hong Kong household members (paired and unpaired)<br>Hong Kong healthy unrelated controls
Mainland China Crohn's disease cases	VS	Mainland China non-Crohn's disease controls (all controls)<br>Mainland China first degree relatives (paired and unpaired)<br>Mainland China healthy household members (paired and unpaired)<br>Mainland China healthy unrelated controls

Supplementary Table S2. Definitions and Examples of Processed Foods

Food	Definition
Processed Dairy	Dairy foods, other than plain milk or natural/Greek yoghurt, which have been modified by the addition of flavours, preservatives, sweeteners or thickeners. Includes, flavoured milk, flavoured yogurt, or milk beverages (except whole milk) (E.g. Moo™ chocolate milk, Dare™ iced coffee
Processed meats or seafood	Meat, chicken, or seafood products that have been preserved or flavoured using additives. Includes sausages, burgers, deli meats, chicken nuggets, chicken schnitzel, fish fingers, crab cakes
Processed grains	Highly refined grain foods that have been preserved, flavoured or texture modified ith the addition of dietary additives. Includes supermarket bread, crackers, store-bought pasta, instant noodles
Processed fruits	Fruits that have been preserved or sweetened with dietary additives, includes frozen, canned, pickled, dried fruits
Processed vegetables	Vegetables that have been preserved or sweetened with dietary additives, includes. frozen, canned, pickled vegetables
Fast food	Over the counter, immediately cooked, ultra-processed foods purchased at chain restaurants, such as McDonald's, Hungry Jacks, KFC, and Pizza Hut
Soft drink	Sugar-sweetened, additive-containing beverages, includes Coke™, Sprite™, Gatorade™
Processed snacks	Packaged, energy-dense, nutrient-poor foods, typically consumed between meals, that have been modified with dietary additives. Includes crackers, sweets, biscuits, muesli bars

Supplementary Table S3. Proportion of Individuals who consumed processed foods on a weekly basis in Early Life

Question	**AUS** (n=179)	**HK** (n= 141)	**CHINA** (n=190)	**All** Crohn's Disease Cases (n=273)	**All** Non-Crohn's Disease Controls (n=237)	**All** HHM (n=116)	**All** FDR (n=81)	**All** Healthy Unrelated Controls (n=92)	**AUS** Crohn's Disease Cases (n=100)	**AUS** All Non-Crohn's Disease Controls (n=79)	**AUS** HHM (n=49)	**AUS** FDR (n=27)	**AUS** Healthy Unrelated Controls (n=20)	**HK** Crohn's Disease Cases (n =94)	**HK** All Non-Crohn's Disease Controls (n = 47)	**HK** HHM (n=39)	**HK** FDR (n=21)	**HK** Healthy Unrelated Controls (n=22)	**CHINA** Crohn's Disease Cases (n=79)	**CHINA** All Non-Crohn's Disease Controls (n =111)	**CHINA** HHM (n=28)	**CHINA** FDR (n=33)	**CHINA** Healthy Unrelated Controls (n=50)
Breastfed vs formula fed	82.4†#	51.3	94.7§	73.3	81.0 ^	81.0	79.0	82.6	84.0†	80.5	81.6	74.1	90.0	46.8§	57.8	66.7	64.0	40.9	91.1	97.3	100.0	93.9	98.0
Bottled food when weaned	23.2†#	10.2	5.3	14.0	11.4	14.7	11.0	7.6	23.2	23.2	22.4	25.0	15.0	12.9§	6.3	7.7	4.0	4.5	3.8	6.3	10.7	3.0	6.0
Foods from convenience stores	7.7	5.1	0.5§	5.9	2.8	3.4	3.7	1.1	8.0#	7.2	6.0	7.1	5.0	7.5	1.6	2.6	4.0	0.0	1.3	0.0	0.0	0.0	0.0
Ate homegrown foods 4-12 months	30.6†#	8.2	64.2§	29.3+	40.9^	34.2	42.72	47.83	31.0†#	30.1	32.0	32.1	30.0	5.3§	12.5	12.8	12.0	9.1	55.7	70.3^	67.9	69.7	72.0[3]
Ate homegrown foods 1-5 years	34.4†#	9.5	66.3§	30.8+	44.0^	36.8	43.92	53.33	32.0†#	37.3	36.0	35.7	50.0	6.4§	14.1	12.8	8.0	13.6	58.2	72.0^	71.4	72.7	72.0
Ate homegrown foods 5-10 years	35.5†#	9.5	62.6§	31.1+	41.6^	35.9	40.2	50.03	34.0†#	37.3	36.0	32.1	50.0	7.4§	12.5	10.3	8.0	13.6	55.7	67.6	71.4	66.7	66.0
Ate homegrown foods 10-18 years	34.4#†	8.2	54.7§	27.5+	38.5^	35.0	39.02	42.43	30.0†#	39.8	34.0	42.9	50.0	7.4§	9.4	10.3	8.0	4.5	48.1	59.5	71.4[1]	54.5	56.0
Ate processed dairy 4-12 months	9.9†#	18.4	3.2§	11.0+	8.6	13.7	2.42	7.6	6.1+†	14.5	16.0[1]	3.6	20.0	22.3§	12.5	15.4	8.0	9.1	3.8	2.7	7.1	0.0	2.0
Ate processed dairy 1-5 years	23.5†#	36.1	5.8§	25.3+	16.6^	23.9	11.02	12.13	22.0†#	25.3	24.0	21.4	30.0	41.5+§	28.1	35.9	20.0	18.2	10.1	2.7^	7.1	0.0	2.0
Ate processed dairy 5-10 years	41.5	49.4	13.2§	39.2+	29.2^	37.6	28.0	19.63	40.0#	43.4	36.0	39.3	55.0	52.1+§	45.3	59.0	48.0	22.7[3]	22.8+	6.3^	17.9	6.1[2]	4.0[3]
Ate processed dairy 10-18 years	47.8†#	71.5	23.7§	51.8+	43.6	51.3	41.5	35.93	45.5†	50.6	46.0	46.4	65.0	75.5§	65.6	82.1	76.0	40.9[3]	31.6	18.0^	10.7	12.1[2]	22.0
At processed meat 4-12 months	10.9	6.3	2.1	8.1	4.1^	6.0	2.4	3.3	11.0†#	10.8	10.0	7.1	10.0	9.6	1.6	0.0	0.0	4.5	2.5	1.8	-	0.0	0.0
At processed meat 1-5 years	48.1†#	36.7	4.7§	33.3+	24.7^	34.2	19.52	17.43	48.0#	48.2	46.0	42.9	45.0	38.3§	34.4	38.5	20.0	31.8	8.9	1.8^	7.1	0.0	0.0[3]
At processed meat 5-10 years	74.3	65.8	11.6§	56.4+	44.3^	53.8	41.52	34.83	77.0#	71.1	70.0	71.4	70.0	68.1§	62.5	64.1	56.0	63.6	16.5	8.1	10.7	6.1	8.0
At processed meat 10-18 years	82.5	83.5	26.3§	70.3+	58.1^	67.5	54.92	48.93	83 .0#	81.9	80.0	78.6	85.0	83.0§	84.4	89.7	80.0	81.8	39.2+	17.1^	14.3[1]	15.2[2]	20.0[3]
Ate processed grains 4-12 months	25.1†#	10.8	0.5§	13.9	9.7	11.1	9.8	7.7	26.0†#	24.1	18.0	25.0	25.0	12.8§	7.8	7.7	8.0	9.1	0.0	0.9	3.6	0.0	0.0
Ate processed grains 1-5 years	68.9#	57.0	5.8§	49.8+	36.8^	48.7	32.92	25.03	71.0†	66.3	64.0	60.7	65.0	59.6 §	53.1	59.0	52.0	45.5	11.4+	1.8^	7.1	0.0[2]	0.03
Ate processed grains 5-10 years	82.0	79.1	14.7§	65.2+	51.9^	64.1	50.02	38.03	83.0	80.7	80.0	85.7	75.0	79.8§	78.1	82.1	72.0	77.3	25.3+	7.2^	10.7	6.1[2]	6.0
Ate processed grains 10-18 years	89.6	93.0	28.9§	77.7+	63.2^	73.5	62.22	51.13	89.0	90.4	92.0	96.4	80.0	95.7§	89.1	89.7	84.0	90.9	41.8+	19.8^	17.9 [1]	18.2[2]	22.0[3]
Ate processed fruit 4-12 months	22.0 †#	6.3	1.6§	12.9+	6.2^	8.5	4.92	4.33	26.3†#	16.9	16.0	14.3	10.0	8.5§	3.1	2.6	0.0	4.5	1.3	1.8	3.6	0.0	2.03
Ate processed fruit 1-5 years	47.3†#	19.6	6.8§	27.6	21.0	26.5	18.3	16.33	49.5†#	44.6	38.0	39.3	50.0	21.3§	17.2	20.5	20.0	13.6	7.6	6.3	14.3	9.1	4.03
Ate processed fruit 5-10 years	58.2 †#	38.6	11.1§	37.9+	34.7	43.6	36.6	21.73	56.6#	60.2	54.0	67.9	60.0	39.4§	37.5	46.2	40.0	27.3	12.7	9.9	21.4	0.0	4.03
Ate processed fruit 10-18 years	65.7	56.3	15.9§	49.4+	43.1	52.1	39.5	34.83	64.3#	67.5	62.0	67.9	70.0	57.4§	54.7	64.1	60.0	45.5	21.5	11.8	17.9	0.0[2]	16.0[3]
Ate processed veg 4-12 months	19.7†#	3.8	71.1§	22.3+	39.9^	29.9	40.22	52.23	14.0#	26.5^	24.0	17.9	25.0	5.3 §	1.6	0.0	0.0	4.5	53.2+	83.6^	82.1[1]	84.8[2]	84.0
Ate processed veg 1-5 years	43.7†#	20.3	87.9§	41.4+	53.6^	47.9	52.4	62.03	43.0#	44.6	40.0	32.1	50.0	21.3§	18.8	23.1	20.0	13.6	63.3+	91.0^	96.4[1]	97.0[2]	88.0
Ate processed veg 5-10 years	56.8†#	42.4	91.6§	58.6+	64.9	59.0	62.2	75.03	58.0#	55.4	52.0	42.9	60.0	44.7§	39.1	41.0	36.0	40.9	75.9+	96.4^	96.4[1]	100.0[2]	96.0
Ate processed veg 10-18 years	63.9	58.9	100.0§	68.9	73.5	70.9	69.5	80.43	66.0#	61.4	60.0	42.9	65.0	57.4§+	60.9	66.7	64.0	59.1	86.1	95.5^	86.1	93.9	96.0
Ate fast food 4-12 months	3.3	3.2	0.0§	3.7+	0.3^	0.9	0.0	0.0	5.0#	1.2	2.0	0.0	0.0	5.3§	0.0	0.0	0.0	0.0	0.0	0.0	0.0	0.0	0.0
Ate fast food 1-5 years	14.8†#	24.1	1.6§	17.2+	8.6^	12.0	8.5	4.33	16.0#	13.3	12.0	10.7	10.0	29.8§^	15.6	23.5	16.0	9.1[C]	3.8	0.0^	0.0	0.0	0.0
Ate fast food 5-10 years	32.2†#	48.7	6.8§	35.9+	20.3^	24.8[1]	22.02	13.03	38.0#	25.3	16.0[A]	25.0	30.0	55.3§*	39.1	52.9	44.0	22.7[C3]	10.1	4.5	7.1	6.1	2.0[3]
Ate fast food 10-18 years	54.6†#	79.7	13.2§	55.7+	40. 9^	47.9	42.7	30.43	56.0#	53.0	48.0	53.6	45.0	85.1§+	71.9^	62.5	80.0	68.2	20.3	8.^	10.7	6.1	8.0[3]
Drank soft drinks 4-12 months	3.3	5.7	0.0§	4.4	1.0^	0.9	1.2	1.1	9.0	15.7	12.0	7.1	25.0	12.8§	1.6	0.0	0.0	4.5	0.0	0.0	0.0	0.0	0.0
Drank soft drinks 1-5 years	17.5†#	33.9	2.6§	22.0	16.6	21.4	15.9	11.03	50.0†#	49.4	52.0	46.4	40.0	59.6 §	50.0	59.0	56.0	36.4	3.8	1.8	3.6	0.0	2.0
Drank soft drinks 5-10 years	45.4†#	69.0	13.7§	49.5+	34.0^	43.6	31.72	23.93	75.0†#	72.3	70.0	64.3	75.0	80.9§	68.8	76.9	72.0	59.1	24.1+	6.3^	10.7	3.0[2]	26.0[3]
Drank soft drinks 10-18 years	71.0†#	91.1	29.5§	68.1+	60.5	71.8	61.0	45.73	85.0	86.7	86.0	78.6	90.0	89.4§	87.5	87.2	84.0	90.9	41.8+	20.7^	21.4	15.2[2]	24.0[3]
Ate processed snacks 4-12 months	12.0	8.2	3.2	8.8	5.8	6.8	3.7	6.5	4.0	2.4	2.0	3.6	0.0	8.5§	1.6	0.0	0.0	4.5	3.8	2.7	7.1	3.0	0.0
Ate processed snacks 1-5 years	49.7#	55.7	16.8§	46.2+	35.1^	45.3	35.4	21.73	17.0#	18.1	16.0	17.9	15.0	42.6§	35.9	41.0	40.0	27.3	25.3	10.8^	14.3	12.1	8.0[3]
Ate processed snacks 5-10 years	73.8#	75.9	26.8§	67.8+	49.5^	59.8	47.62	38.03	46.0#	44.6	42.0	42.9	40.0	74.5§	60.9	69.2	60.0	50.0[C]	43.0+	15.3^	17.9[1]	15.2[2]	14.0[3]

Ate processed snacks 10-18 years	85.8[#]	88.6	43.7[§]	79.9+	65.3^	75.2	59.82	57.63	65.0[#]	78.3^	86.0	89.3[2]	55.0	93.6[§]	87.5	89.7	92.0	86.4	62.0+	30.6^	39.3[1]	24.2[2]	30.0[3]

Difference across regions OR difference across Crohn's Disease participants across regions:

† Australia different to Hong Kong

Australia different to China

§ Hong Kong different to China

^ Crohn's Disease participants different to non- Crohn's Disease participants combined

+ Difference across participant types

Post-Hoc (where +, is significant): 1 – Crohn's Disease different to Household member (unpaired); 2 - Crohn's Disease different to First Degree Relatives (unpaired); 3- Crohn's Disease different to Healthy Controls (unpaired)

a - Crohn's Disease different to Household member (paired); b - Crohn's Disease different to First Degree Relatives (paired); c- Crohn's Disease different to Healthy Controls (paired)

AUS – Australia, HK – Hong Kong, HHM – Healthy Household Members, FDR – First Degree Relatives

In Australia 17 FDRs were also analysed as Household members; In Hong Kong 35 FDRs were also analysed as Household members

Supplementary Table S4. Median annual intake of additives calculated based on responses to the survey on intake of processed foods consumed in the past 12 months

Intake median (mg)	Australia (n=180)	Hong Kong (n= 142)	China (n=190)	**All** Crohn's Disease Cases (n=274)	**All** Non-Crohn's Disease Controls (n=238)	**All** Healthy HHM (n=118)	**All** FDR (n=81)	**All** Healthy Unrelated Controls (n=91)	**AUS** Crohn's Disease Cases (n=100)	**AUS** All Non-Crohn's Disease Controls (n=62)	**AUS** HHM (n=50)	**AUS** All FDR (n=28)	**AUS** Healthy Unrelated Controls (n=19)	**HK** Crohn's Disease Cases (n=95)	**HK** All Non-Crohn's Disease Controls (n=82)	**HK** HHM (n=39)	**HK** All FDR (n=21)	**HK** Healthy Unrelated Controls (n=22)	**CHINA** Crohn's Disease Cases (n=79)	**CHINA** All Non-Crohn's Disease Controls (n =118)	CHINA HHM (n=30)	**CHINA** All FDR (n=31)	**CHINA** Healthy Unrelated Controls (n=50)
**Total Additive Intake**	1.9E+6 (1.8E+6)	1.1E+6 (1.2E6)	4.0+E5 (4.9E+5)	1.2E+6 (1.9E+6)	8.2E+5^ (1.4E+6)	1.0E+6 (1.8E+6)	7.2E+5[b2] (1.4E+6)	7.1E+5[3] (9.8+E+5)	2.1E+6+[†#]* (1.3E+6)	1.7E+6^ (2.1E+5)	1.8E+6 (3.0E+6)	1.5E+6[2] (1.3E+6)	1.6E+6 (1.2E+6)	1.2E+6 (1.3E+6)	9.8E+6 (1.1E+5)	9.9E+5[1] (1.2E+6)	8.7E+5 (1.0E+5)	1.0E+6 (1.1E+5)	4.7E+5+[§] (-8.0E+5)	3.7E+5 (-3.9E+5)	3.3E+5 (-3.9E+5)	3.7E+5[2] (-3.5E+5)	3.9E+5 (-4.9E+5)
**Total Artificial Sweetener Intake**	2.0E+5[†#] (2.6E+5)	8.1E+4 (1.1E+3)	2.0E+5[§] (-2.7E+5)	9.5E+4+ (1.8E+5)	4.8E+4^ (1.4E+5)	7.3E+5 (1.9E+5)	4.5E+4[2] (1.2E+5)	3.6E+4[3] (7.4E+4)	2.0E+5[#]* (2.8E+5)	2.0E+5** (2.5E+5)	2.3E+5 (2.5E+5)	1.9E+5[2] (2.8E+5)	1.3E+5 (1.6E+5)	9.6E+4+ (1.2E+5)	5.2E+4^ (9.2E+4)	4.8E+4[a1] (9.2E+4)	3.1E+4[b] (8.9E+4)	7.4E+4 (2.7E+4)	2.9E+3+ (-3.7E+4)	1.8E+4 (-2.8E+4)	1.4E+4 (-2.0E+4)	3.0E+4 (-3.1E+4)	1.6E+4 (-2.1E+4)
**Total Emulsifiers Intake**	1.7E+5[†#] (1.9E+6)	1.1E+6 (1.1E+6)	1.7E+6[§] (-1.9E+6)	1.1E+6+ (1.6E+6)	7.7E+5^ (1.2E+6)	9.2E+5 (1.6E+6)	6.6E+5[b2] (1.2E+6)	6.4E+5[3] (8.9E+5)	1.9E+6[†#]* (2.3E+6)	1.4E+6*^ (1.6E+6)	1.5E+6 (2.1E+6)	1.4E+6[b2] (1.3E+6)	1.4E+6 (1.8E+6)	1.1E+4 (1.2E+6)	9.3E+5 (1.1E+6)	9.7E+5 (1.5E+6)	8.0E+5[b] (1.3E+6)	9.5E+5 (8.5E+4)	4.6E+5+[§] (-7.1E+5)	3.6E+5^ (-3.7E+5)	3.0E+5[a] (-3.7E+5)	3.4E+5[2] (-3.2E+5)	3.3E+5 (-4.4E+5)
Aluminosilicates	1.2E+3[#] (4.3E+3)	1.5E+3 (3.4E+3)	0.0E+0[§] (4.8)	8.3+E3+ (3.5E+3)	1.4E+5^ (1.6E+3)	4.9E+2 (2.5E+3)	4.8E+1[b2] (7.9E+2)	1.2E+0[3] (1.2E+3)	1.2E+3[†#]* (4.3E+3)	1.2E+3 (4.1E+3)	1.2E+3 (3.9E+3)	7.4E+2[2] (3.0E+3)	1.3E+3 (8.2E+3)	1.7E+3+ (3.5E+3)	5.5E+2 (3.3E+4)	9.0E+2 (3.3E+3)	4.4E+2[b] (2.1E+3)	8.1E+2 (3.9E+3)	7.0E-1+[§] (-8.4E+2)	0.0E+0 (-5.2E4)	0.0E+0[1] (0.0E+0)	0.0E+0[b2] (-1.8E+0)	0.0E+0[3]
Aspartame	9.3E+4[†#] (1.3E+5)	4.3E+4 (6.4E+4)	1.1E+4[§] (-1.7E+4)	4.9E+4+ (8.2E+4)	2.5E+4^ (7.4E+4)	3.5E+4[a] (1.0E+)	2.4E+4[2] (5.7E+5)	2.1E+4[3] (4.6E+4)	9.5E+4[†#]* (1.4E+5)	8.9E+4* (1.3E+5)	1.1E+5 (1.4E+5)	8.7E+4[2] (1.5E5)	6.0E+4 (5.4E+4)	5.4E+4+ (6.7E+4)	2.8E+4^ (4.9E+4)	2.9E+4[a1] (4.6E+4)	1.9E+4 (5.2E+4)	3.6+4 (4.4E+4)	1.1E+4+[§] (-2.2E+4)	9.7E+3^ (-1.5E+4)	8.0E+3 (-1.2E+4)	1.6E+4 (-1.4E+4)	4.9E+5 (-1.4E+4)
Carboxymethyl-cellulose	7.7E+5[†#] (1.1E+6)	4.5E+5 (6.5E+5)	2.0E+5[§] (-2.3E+5)	4.5E+6+ (8.2+6)	3.0E+5^ (5.6E+5)	3.4E+5 (8.0E+5)	2.3E+5[b2] (5.5E+5)	2.6E+5[3] (4.6E+5)	9.0E+5[†#]* (1.2E+6)	6.6E+5* (9.5E+5)	7.1E+5 (1.2E+6)	6.1E+5[2] (6.6E+5)	6.2E+5 (1.1E+6)	5.0E+5 (7.0E+5)	3.7E+5[§] (7.0E+5)	4.0E+5 (6.9E+5)	3.0E+5[b] (5.4E+5)	4.1E+5 (5.1E+5)	2.2E+5+[§] (-3.4E+5)	2.0E+5 (-1.8E+5)	1.6E+5[a] (-1.8E+5)	1.9E+5 (-1.4E+5)	2.0E+5 (-2.3E+5)
Carrageenan	5.0E+5[†#] (6.1E+5)	3.1E+5 (4.3E+5)	9.7E+4[§] (-1.6E+5)	3.0E+5+ (5.0E+5)	2.0E+5^ (3.8E+5)	2.7E+5 (4.5E+5)	1.5E+5[b2] (4.0E+5)	1.8E+5[3] (2.5E+5)	5.1E+5[†#]* (7.0E+5)	4.5E+5* (5.5E+5)	4.5E+5 (7.6E+5)	4.05E+5 (4.5E+5)	5.0E+5 (4.6E+5)	3.2E+5 (4.4E+5)	3.0E+5 (3.5E+5)	3.1E+5 (4.1E+5)	3.0E+5 (4.6E+5)	3.1E+5 (1.9E+5)	1.5E+5+[§] (-2.3E+5)	8.2E+4^ (-1.2E+5)	8.2E+4[1a] (-1.2E+5)	7.7E+4[2] (-9.5E+4)	8.6E+4[3] (-1.4E+5)
Polysorbate-80	3.8E+5[†#] (3.1E+5)	2.6E+5 (3.8E+5)	7.3E+3[§]	2.7E+5+ (4.3E+5)	1.6E+5^ (2.7E+5)	2.0E+5[a1] (3.2E+5)	1.5E+5[b2] (2.5E+5)	1.4E+5[3] (2.0E+5)	4.2E+5+* (3.9E+5)	3.1E+5*^ (2.5E+5)	3.4E+5[a1] (3.4E+5)	2.8E+5[b2] (2.1E+5)	2.9E+5[3c] (4.0E+5)	2.7E+5 (4.2E+5)	2.5E+5 (3.1E+5)	2.3E+5 (3.4E+5)	2.3E+5[b] (3.1E+5)	2.8E+5 (2.3E+5)	8.4E+4[§] (-1.4E+5)	7.1E+4 (-8.1E+4)	6.2E+4[1a] (-8.5E+4)	6.5E+4 (-5.2E+4)	7.6E+4 (-9.6E+4)
Saccharin	3.6E+4[†#] (5.0E+4)	1.3E+4 (2.0E+4)	2.8E+3[§] (-3.8E+3)	1.7E+4+ (3.2E+4)	7.6E+3^ (2.2E+4)	1.1E+4 (4.0E+4)	6.7E+4[b2] (1.4E+4)	5.0E+3[3] (1.1E+4)	3.5E+4[†#]* (4.7E+4)	3.7E+4* (5.0E+4)	4.3E+4 (4.9E+4)	2.2+4[2b] (5.6E+4)	2.3E+4 (4.6E+4)	1.7E+4+ (2.3E+4)	1.0E+4^ (1.3E+4)	9.9E+31 (1.5E+4)	8.1E+3[b] (1.1E+4)	1.1+4 (9.5E+3)	2.9E+3[§] (-5.6E+3)	2.8E+3 (-3.0E+3)	2.5E+3 (-3.0E+3)	3.8E+3 (-3.5E+3)	2.5E+3(- 3.0E+3)
Sucralose	6.0E+4[†#] (8.5E+4)	1.9E+4 (2.8E+4)	4.3E+3[§] (-7.5E+3)	2.3E+4+ (5.3E+4)	1.2E+4^ (3.7E+4)	1.8E+4[a] (5.8E+4)	1.4E+4[b2] (3.3E+4)	9.0E+3[3] (2.0E+4)	5.8E+4[†#]* (9.2E+4)	6.1E+4* (7.3E+4)	6.9E+4 (9.4E+5)	4.6E+4[2] (8.2E+4)	4.2E+4 (6.3E+4)	2.4E+4+ (3.6E+4)	1.2E+4^ (2.3E+4)	1.0E+4[1] (2.5E+4)	6.6E+3[b] (2.1E+4)	1.6+4 (1.7E+4)	4.3E+3+[§] (-7.4E+3)	4.4E+3 (-7.6E+3)	3.3E+3 (-4.6E+3)	8.5E+3 (-1.4E+4)	3.9E+3(- 7.1E+3)
Sulphites	2.0E+4[†#] (3.4E+4)	2.9E+3 (4.3E+3)	1.9E+3[§] (2.2E+3)	4.0E+3+ (1.4E+4)	3.1E+3^ (1.1E+4)	3.3E+3[a] (2.1E+4)	3.7E+3 (9.9E+3)	2.7E+3[3] (6.1E+3)	1.9E+4[†#]* (2.8E+4)	2.1E+4* (4.1E+4)	2.4E+4 (6.8E4)	2.0E+4[2] (3.7E+4)	1.7E+4 (3.3E+4)	3.4E+3+^[§] (6.2E+3)	2.1E+3 (3.5E+4)	1.8E+3 (2.7E+3)	1.5E+3 (2.8E+3)	3.1E+3 (6.5E+3)	1.8E+3+[§] (-2.3E+3)	1.9E+3 (-2.2E+3)	1.4E+3 (-1.8E+3)	2.6E+3[b] (-6.2E+3)	1.9E+3 (-1.8E+3)
Titanium Dioxide	1.9E+4[#] (4.9E+4)	4.8E+3 (2.3E+4)	3.1E+3 (2.4E+3)	8.2E+4+ (3.2E+4)	8.1E+2^ (1.4E+4)	.5E+3 (2.7E+4)	5.8E+2[b2] (1.0E+4)	2.2E+2[3] (5.2E+3)	2.2E+4[†#]* (5.0E+4)	1.6E+4** (4.2E+4)	1.8E+4 (3.7E+4)	1.2E+4[b2] (2.2E+4)	1.6E+4 (5.9E+4)	8.8E+3+[§] (2.8E+4)	1.4E+3^ (9.5E+4)	1.5E+3[1] (2.7E+4)	6.0E+2[b] (8.1E+3)	2.1E+3[c3] (5.5E+3)	4.6E+1+[§] (-1.4E+3)	2.0E+0^ (-1.6E+1)	0.0E+0[1] (-4.8E+1)	8.0E+0 (-1.6E+2)	4.4E+5[3] (-1.3E+2)

Difference across regions OR difference across Crohn's Disease participants across regions:

[†] Australia different to Hong Kong

[#] Australia different to China

[§] Hong Kong different to China

^ All non-Crohn's different to Crohn's (Mann-Whitney, unpaired)

+Difference between groups (Kruskal-Wallis, unpaired);

Post-Hoc (where +, is significant): [1] Household member different to Crohn's (unpaired); [2] Household member different to Crohn's (unpaired); [3] Unrelated control different to Crohn's (unpaired); [3] Unrelated controls different to Crohn's (Mann-Whitney, unpaired);

[a] Household member different to Crohn's (paired); [a] Household member different to Crohn's (Wilcoxon-Sign, paired); [b] First degree relative different to Crohn's (Wilcoxon, sign paired); [c] Unrelated control different to Crohn's (Mann-Whitney, unpaired

AUS – Australia, HK – Hong Kong, HHM – Healthy Household Members, FDR – First Degree Relatives

In Australia 17 FDRs were also analysed as Household members; In Hong Kong 35 FDRs were also analysed as Household members

Supplementary Table S5: Median recent intake of additives calculated based on maximal permitted level of additives present in foods recorded in 3-day food diaries

	**AUS** All Subjects (n=176)	**HK** All Subjects (n=140)	**All** Crohn's Disease Cases (n=193)	**All** Non-Crohn's Disease Controls (n=123)	**All** Healthy Household Members (n=84)	**All** First Degree Relatives (n=51)	**All** Healthy Unrelated Controls (n=40)	**AUS** Crohn's Disease Cases (n=99)	**AUS** All Non-Crohn's Disease Controls (n=77)	**AUS** Healthy Household Members (n=49)	**AUS** First Degree Relatives (n=27)	**AUS** Healthy Unrelated Controls (n=18)	**HK** Crohn's Disease Cases (n=94)	**HK** Non-Crohn's Disease Participant's (n=46)	**HK** Healthy Household Members (n=35)	**HK** First Degree Relatives (n=24)	**HK** Healthy Unrelated Controls (n=22)
**Total Additive Intake**	17 (24) [0-93]	0 (20) [0-98]	0 (25) [0-98]	0 (19) [0-23]	0 (22) [0-93]	0 (18) [2, b] [0-56]	0 (15) [0-57]	0 (27) [0-70]	0 (21) [0-93]	0 (21) [0-93]	0 (20) [0-56]	0 (17) [0-39]	0 (19) [0-27]	0 (15) [0-68]	0 (19) [1a] [2-69]	0 (13) [2] [0-51]	0 (21) [3-57]
**Total Artificial Sweetener Intake**	0 (0) [0-2295]	0 (0) [0-9.55E3]	0 (0) [0-2295]^	0 (0) [0-9.55E3]	0 (4)[1] [0-540]	0 (0)[2] [0-1575]	0 (0) [0-9.55E3]	0 (0) [0-2295]	0 (0) [0-1575]	0 (0) [0-540]	0 (0) [0-1575]	0 (0) [0-517]	0 (0) [0-335]+	0 (0) [0-9.55E3]	0 (4) [0-21][1a]	0 (0)[2] [0]	0 (0) [0-9.55E3]
**Total Emulsifier Intake**	0 (0) [0-6810]	0 (0) [0-25005]	0 (5) [0-25005]	0 (79) [0-3960]	0 (3) [0-930]	0 (0)[2] [0-440]	0 (87) [0-3960]	0 (18) [0-6810]	0 (3) [0-960]	0 (34) [0-930]	0 (0) [0-444]	0 (240) [0-960]	0 (0) [0-25005]+	0 (0) [0-3960]	0 (0)[1a] [0-230]	0 (0)[2] [0]	0 (75) [0-3960]
Aluminosilicates	0 (0) [0]	0 (0) [0]	0 (0) [0]	0 (0) [0]	0 (0) [0]	0 (0) [0]	0 (0) [0]	0 (0) [0]	0 (0) [0]	0 (0) [0]	0 (0) [0]	0 (0) [0]	0 (0) [0]	0 (0) [0]	0 (0) [0]	0 (0) [0]	0 (0) [0]
Aspartame	0 (0) [0-765]***	0 (0) [0-300]	0 (0) [0-765]	0 (0) [0-525]	0 (0) [0-180]	0 (0) [0-526]	0 (0) [0-203]	0 (0) [0-765]**	0 (0) [0-525]*	0 (0) [0-180]	0 (0) [0-525]	0 (0) [0-292]	0 (0) [0-300]	0 (0) [0]	0 (0) [0]	0 (0) [0]	0 (0) [0]
Carboxymethylcellulose	0(0) [0-5675]	0 (0) [0-4950]	0 (0) [0-5675]	0 (0) [0-3300]	0 (0) [0-425]	0 (0) [0-370]	0 (5) [0-3300]	0 (0) [0-5675]	0 (0) [0-800]	0 (0) [0-425]	0 (0) [0-370]	0 (0) [0-800]	0 (0) [0-4950]+	0 (0) [0-3300]	0 (0) [0]	0 (0) [0]	0 (13) [0-3300]
Carrageenan	0 (0) [0-1135]	0 (0) [0-1065]	0(0) [0-1135]	0 (0) [0-960]	0 (4)[1] [0-230]	0 (0) [0-350]	0 (0) [0-960]	0(0) [0-1135]	0 (0) [0-960]	0 (0) [0-199]	0 (0) [0-350]	0 (0) [0-960]	0 (0) [0-10651]	0 (0) [0-660]	0 (0)[1] [0-230]	0 (0)[2] [0]	0 (1) [0-660]
Polysorbate-80	0 (0) [0-1215]	0 (0) [0 -25000]	0 (0) [0-25000]	0 (0) [0-930]	0 (0) [0-930]	0 (0) [0]	0 (0) [0]	0 (0) [0-1215]	0 (0) [0-930]	0 (0) [0-930]	0 (0) [0]	0 (0) [0]	0 (0) [0-255000]	0 (0) [0]	0 (0) [0]	0 (0) [0]	0 (0) [0]
Saccharin	0(0) [0]	0 (0) [0-15]	0 (0) [0-15]	0 (0) [0]	0 (0) [0]	0 (0) [0]	0 (0) [0]	0 (0) [0]	0 (0) [0]	0 (0) [0]	0 (0) [0]	0 (0) [0]	0 (0) [0-15]	0 (0) [0]	0 (0) [0]	0 (0) [0]	0 (0) [0]
Sucralose	0.0 (0) [0-149]	0 (0) [0-9.55E3]	0 (0) [0-142]	0 (0) [0-9.55E3]	0 (0) [0]	0 (0) [0]	0 (0) [0-9.55E3]	0 (0) [0-142]	0 (0) [0-18]	0 (0) [0]	0 (0) [0]	0 (0) [0-15]	0 (0) [0-15]	0 (0) [0-9.55E3]	0 (0) [0]	0 (0) [0]	0 (0) [0-9.55E3]
Sulphites	0 (0) [0-320]	0 (0) [0-20]	0 (0) [0-320]	0 (0) [0-75]	0 (0) [0-13]	0 (0) [0-20]	0 (5) [0-14]	0 (0) [0-2295]	0 (0) [0-75]	0 (0) [0-13]	0 (0) [0-4]	0 (0) [0-75]	0 (0) [0-20]	0 (0) [0-20]	0 (0) [0]	0 (5) [0-20]	0 (0) [0-20]
Titanium Dioxide	0 (0) [0-627]*	0 (0) [0-400]	0 (0) [0-672]	0 (0) [0-600]	0 (0) [0-102]	0 (0) [0-240]	0 (0) [0-600]	0 (0) [0-672]	0 (0) [0-600]	0 (0) [0-192]	0 (0) [0-240]	0 (0) [0-600]	0 (0) [0-400]	0 (0) [0]	0 (0) [0]	0 (0) [0]	0 (0) [0]

*Difference between countries (P<0.05);
** Difference between countries (P<0.001);
^ All non-Crohn's different to Crohn's (Mann-Whitney, unpaired);
+Difference between groups (Kruskal-Wallis, unpaired); Post-Hoc (where +, is significant):
[1] Household member different to Crohn's (unpaired); [2] Household member different to Crohn's (unpaired); [3] Unrelated control different to Crohn's (unpaired); [3] Unrelated controls different to Crohn's (Mann-Whitney, unpaired);
[a] Household member different to Crohn's (paired); [a] Household member different to Crohn's (Wilcoxon-Sign, paired); [b] First degree relative different to Crohn's (Wilcoxon, sign paired); [c] Unrelated control different to Crohn's (Mann-Whitney, unpaired)

AUS – Australia, HK – Hong Kong
In Australia 17 FDRs were also analysed as Household members; In Hong Kong 35 FDRs were also analysed as Household members

Supplementary Table S6: Median Recent Intake of Energy, Nutrients and Alcohol, Estimated Based on Foods Recorded in 3-Day Food Diaries

Nutrient Median (IQR)	**AUS** All Subjects (n=178)	**HK** All Subjects (n=154)	**All** Crohn's Disease Cases (n=193)	**All** Non-Crohn's Disease Controls (n=139)	**All** Healthy Household Members (n=84)	**All** First Degree Relatives (n=51)	**All** Healthy Unrelated Controls (n=40)	**AUS** Crohn's Disease Cases (n=99)	**AUS** All Non-Crohn's Disease Controls (n=79)	**AUS** Healthy Household Members (n=49)	**AUS** First Degree Relatives (n=27)	**AUS** Healthy Unrelated Controls (n=18)	**HK** Crohn's Disease Cases (n=94)	**HK** Non-Crohn's Disease Participant's (n=60)	**HK** Healthy Household Members (n=35)	**HK** First Degree Relatives (n=24)	**HK** Healthy Unrelated Controls (n=22)
DII	0.2152* (2.34)	2.6308 (1.75)	1.4435 (3.12)	0.7582 ^ (3.04)	0.5576[1] (2.47)	0.5626 [2,b] (-0.171)	1.4479 (3.49)	-0.0659 (2.22)	-0.3421 (2.26)	-0.2322 (2.28)	-0.7625 [2] (2.21)	-0.4541 (2.44)	2.7051 (1.56)	2.3441 (1.96)	2.2204 [1,a] (2.10)	2.2828 [2,b] (1.98)	2.6952 (2.40)
Energy (kJ)	9212.2** (3209.9)	7950.7 (2545.4)	8667.6 (3382.8)	8553.8 (3394.4)	9334.7 (3357.7)	8091.32 (3164.4)	8056.1 (2689.8)	9429.3** (3321.1)	9111.6** (3372.6)	9632.5 (2832.1)	8553.8 (2405.6)	8940.7 (2664.7)	8162.0 (2566.5)	7705.0 (2579.4)	8513.7 (4414.1)	7496.8 2 (3003.6)	7913.9 (2367.3)
Protein (g)	96.5 (36.8)	90.4 (43.4)	94.4 (42.2)	95.4 (33.7)	98.2 (31.9)	91.8 (39.6)	84.7 (34.8)	96.5 (40.7)	96.5 (33.0)	96.3 (23.0)	97.1 (36.5)	90.5 (35.0)	90.6 (44.3)	89.1 43.5)	107.7 (69.6)	90.5 (33.2)	82.9 (37.9)
Total fat (g)	91.6 ** (40.9)	70.4 (28.8)	78.3 + (36.8)	81.2 (39.8)	97.6 (36.4)	70.5 (41.9)	73.8 (25.8)	89.7** (42.1)	94.0** (40.9)	100.3 (24.9)	81.7 (44.5)	88.3 (35.6)	71.3 (29.5)	68.0 (29.3)	77.7 (65.2)	54.8 (36.7)	71.2 (8.5)
Saturated fat (g)	33.0** (17.7)							32.7** (20.3)	33.1** (15.7)	36.9 (13.7)	26.2 (22.1)	32.0 (17.0)					
Polyunsaturated fat (g)	14.0** (8.3)							14.0** (8.9)	14.3** (7.7)	14.5 (6.6)	14.1 (9.4)	13.3 (8.4)					
Monounsaturated fat (g)	35.3** (18.3)							34.4** (17.9)	35.8** (16.5)	36.8 (17.0)	33.2 (19.0)	32.7 (15.5)					
Carbohydrate (g)	222.7 (87.9)	223.5 (89.2)	225.7 (87.7)	219.2 (88.1)	222.0 (99.4)	213.8[b] (92.6)	219.7 (67.3)	225.7 (94.7)	219.8 (86.1)	222.9 (88.8)	223.8 (90.4)	211.3 (61.6)	225.9 (80.5)	217.9 (100.7)	236.3 (133.0)	213.5 (99.9)	231.6 (105.9)
Sugars (g)	77.2** (53.0)	45.0 (37.6)	58.7 (46.5)	64.4 (54.7)	84.4 (75.7)	56.9 (54.2)	62.8 (47.2)	76.5** (55.3)	77.8** (47.4)	88.1 (58.7)	71.1 (51.8)	77.3 (33.9)	45.9 (37.7)	45.3 (38.9)	57.2 (108.2)	44.6 (28.7)	49.6 (49.2)
Alcohol (g)	0.04** (11.3)	0.0 (0.0)	0.0 (1.34)	0.0 (3.9)	0.0[a] (14.4)	0.0 (0.1)	0.0 (2.0)	0.03** (10.9)	0.04** (13.4)	1.7 (20.0)	0.1 (0.7)	0.5 (27.8)	0.0 (0.0)	0.0 (0.0)	0.0 (0.0)	0.0 (0.0)	0.0 (0.0)
Dietary fibre (g)	22.9** (13.0)	11.0 (7.0)	15.0+ (12.8)	20.1^ (13.9)	21.3 [1] (14.4)	19.0[2, b] (13.7)	16.4 (15.3)	21.9 (13.9)	25.3** (13.8)	23.6 (16.1)	26.2[b] (14.9)	26.6 (13.1)	10.3 (6.6)+	12.1^ (9.5)	9.3[a] (14.5)	13.0[2] (7.4)	12.5[3c] (9.1)
Vitamin B12 (ug)	4.1** (3.1)	2.1 (5.0)	3.7+ (4.5)	3.4 (3.2)	4.3 (3.5)	3.0 (2.9)	2.5 (2.6)	4.1** (3.0)	4.2** (2.5)	4.3 (2.7)	3.6 (2.7)	3.9 (3.1)	2.1 (2.6)	2.2 (6.7)	3.4 (5.5)	2.3 (2.9)	1.6 (1.2)
Total folate (mg)	510.5*** (246.8)	115.0 (102.0)	288.2+ (424.2)	423.1^ (424.9)	487.2[1] (386.8)	378.5 (437.7)	285.7 (395.9)	497.7** (285.3)	536.9** (221.4)	544.7 (327.8)	550.1 (202.4)	479.5 (140.7)	128.9 (106.9)	69.6 (109.7)	123.2 (72.0)	154.9 (89.9)	105.9 (83.1)
Sodium (mg)	2580.3 (1433.1)	2931.36 (1252.1)	2974.0+ (1567.2)	2548.0^ (1160.0)	2639.6[1a] (949.1)	2484.2[2b] (1463.6)	2595.4 (1090.5)	2723.2** (1491.8)	2543.5 (1224.8)	2663.6 (1070.9)	2453.2 (1696.5)	2353.9 (931.5)	2672.9 (1129.)+	3152.5^ (1333.0)	2319.9 [1a] (971.2)	2605.1[2b] (1530.9)	2878.1 (699.5)
Calcium (mg)	824.0** (427.8)	480.0 (333.0)	641.4 (564.2)	662.2 (507)	820.9 (657.1)	618.0 (440.1)	593.9 (516.6)	782.7** (480.3)	848.4** (430.6)	929.0 (586.3)	734.4 (388.1)	825.2 (30.5.2)	453.2 (311.3)	480.3 (431.7)	366.8 (191.9)	514.5 (470.8)	433.2 (279.1)
Iron (mg)	11.3** (4.9)	8.4 (5.5)	10.1 (5.9)	10.5 (5.6)	12.0[1a] (5.4)	10.6 (4.7)	9.1 (4.8)	11.2** (5.2)	11.4** (4.7)	12.1 (5.3)	11.3 (2.7)	10.1 (4.7)	9.1 (6.3)	8.0 (5.5)	12.0 (7.9)	9.6 (7.6)	7.6 (5.1)

*Difference between countries (P<0.05);
** Difference between countries (P<0.001);
^ All non-Crohn's different to Crohn's (Mann-Whitney, unpaired);
+Difference between groups (Kruskal-Wallis, unpaired);
Post-Hoc (where +, is significant): [1] Household member different to Crohn's (unpaired); [2] Household member different to Crohn's (unpaired); [3] Unrelated control different to Crohn's (unpaired); [3] Unrelated controls different to Crohn's(Mann-Whitney, unpaired);
[a] Household member different to Crohn's (paired); [a] Household member different to Crohn's (Wilcoxon-Sign, paired); [b] First degree relative different to Crohn's (Wilcoxon, sign paired); [c] Unrelated control different to Crohn's (Mann-Whitney, unpaired)

AUS – Australia, HK – Hong Kong
21 Hong Kong and 15 Australian FDR included in HHM analysis

Supplementary Table S7. Association Between DII, Additives, Nutrients (based on 3-day food diaries), and Crohn's disease Activity Index (CDAI), C-Reactive Protein (CRP) and Hemoglobin (Hb) in Australia and Hong Kong Crohn's disease Patients

	**All**			**Australia**			**Hong Kong**		
	**CDAI (n=193)**	**CRP (n=191)**	**Hb (n=193)**	**CDAI (n=94)**	**CRP (n=94)**	**Hb (n=94)**	**CDAI (n=99)**	**CRP (n=97)**	**Hb (n=99)**
**DII**	0.12	0.24**	-0.18	0.11	-0.17	0.03	-0.14	0.01	-0.00
**Total Additives**	0.00	0.00	-0.18*	0.03	-0.06	-0.35**	-0.06	0.19	0.02
**Total Artificial Sweeteners**	0.04	0.01	-0.08	0.06	-0.08	-0.14	0.04	-0.04	-0.03
**Total Emulsifier Intake**	0.01	-0.11	-0.08	-0.01	-0.18	-0.12	-0.00	-0.00	-0.01
Aluminosilicates	NA	NA	NA	NA	NA	NA	NA	0.05	-0.05
Aspartame	0.02	-0.04	-0.07	0.06	-0.03	-0.15	0.04	0.10	0.02
Carboxymethylcellulose	0.01	-0.14	-0.03	0.02	-0.11	-0.07	0.01	0.01	0.10
Carrageenan	-0.01	-0.09	-0.09	0.00	-0.07	0.00	-0.05	-0.14	0.09
Polysorbate-80	-0.09	-0.04	0.06	-0.15	-0.13	-0.08	0.04	0.12	-0.01
Saccharin	-0.08	0.03	0.05	NA	-0.00	NA	-0.14	0.13	0.07
Sucralose	-0.09	-0.00	-0.03	-0.14	-0.03	-0.12	-0.04	0.21	**-0.03**
Sulphites	-0.14	-0.19*	0.01	-0.10	-0.04	-0.15	0.00	0.02	0.03
Titanium Dioxide	-0.08	-0.01	-0.04	-0.08	-0.15	-0.11	0.03	-0.04	0.14
**Nutrients**									
Energy	-0.14	-0.09	0.16*	-0.09	-0.06	0.10	-0.105	0.01	0.20
Protein	0.01	-0.00	0.16*	0.14	0.02	0.04	-0.16	-0.06	0.27**
Total fat	-0.15*	-0.18*	0.13	-0.12	-0.06	-0.02	-0.07	-0.07	0.24*
Saturated fat	NA	NA	NA	-0.03	-0.03	0.02	NA	NA	NA
PUFAS	NA	NA	NA	-0.16	0.01	0.04	NA	NA	NA
MUFAS	NA	NA	NA	-0.08	-0.01	-0.07	NA	NA	NA
Carbohydrate	-0.12	-0.08	0.18*	-0.11	-0.10	0.17	-0.11	-0.03	0.20
Sugars	-0.06	-0.17*	0.12	0.14	0.05	0.06	-0.08	-0.09	0.06
Alcohol	-0.17*	-0.13	0.09	-0.16	0.01	0.07	0.07	0.09	-0.02
Dietary Fibre	-0.22*	-0.26**	0.21**	-0.04	-0.03	0.08	-0.17	-0.06	0.21*
Iron	-0.04	-0.06	0.20**	0.10	0.00	0.12	-0.62	0.05	0.23*

*Significant at P <0.05

Supplementary Table S8. Correlation between energy-adjusted DII and current, total additive intake (mg/per kg body weight)

	**Whole cohort**	**AUS**	**HK**	**All** Crohn's Disease	**All**	**All**	**All**	**All**	**AUS** Crohn's Disease Cases	**AUS**	**AUS** Healthy	**AUS**	**AUS** Healthy	**HK** Crohn's Disease Cases	**HK**	**HK** Healthy	**HK**	**HK** Healthy Unrelated
		All Subjects	All Subjects	Cases	Non-Crohn's Disease Controls	Household	First Degree Relatives	Healthy	(n=99)	All Non-Crohn's Disease Controls	Household Members	First Degree Relatives	Unrelated Controls	(n=94)	Non-Crohn's Disease Participant's	Household Members	First	Controls
Total additive intake per kg body weight																		
Spearman's correlation coefficient	0.145	0.170	0.191	0.198	0.016	-0.041	-0.212	0.255	0.261	-0.028	0.123	-0.189	-0.191	0.221	0.094	-0.067	- 0.018	0.185
P-value	**0.015**	**0.032**	**0.035**	**0.012**	0.864	0.737	0.158	0.146	**0.013**	0.821	0.433	0.355	0.513	0.060	0.515	0.739	0.940	0.435

Supplementary Table S9. Differences in Current (3 day) Intake of Energy, Nutrients, and Additives Between Crohn's disease Participants With Normal Versus Elevated CRP, and Normal Versus Low Haemoglobin (Hb)

	All		Australia		Hong Kong		All		Australia		Hong Kong	
**Nutrient and Additive Intake Median (IQR)**	**CRP normal (n=135)**	**CRP elevated (n=56)**	**CRP normal (n=70)**	**CRP elevated (n=24)**	**CRP normal (n=65)**	**CRP elevated (n=32)**	**Hb normal (n=148)**	**Hb low (n=45)**	**Hb normal (n=75)**	**Hb low (n=19)**	**Hb normal (n=73)**	**Hb low (n=26)**
Energy (kJ)	8798.5 (3357.9)	8433.5 (3309.4)	9429.7 (3886.3)	8446.4 (10358.6)	8162.0 (2397.7)	8177.4 (4770.0)	8449.6 (3094.3)	8752.3 (4124.9)	9348.4 (3436.0)	9994.5 (12492.6)	8162.0 (2589.7)	7991.1 (3325.5)
Protein (g)	94.4 (39.5)	93.0 (53.6)	95.9 (44.1)	92.7 (54.6)	91.4 (36.7)	93.0 (55.5)	94.2 (43.0)	97.1 (45.2)	92.5 (43.0)	102.3 (27.9)	94.4 (42.5)	84.0 (43.9)
Total fat (g)	78.75 (40.18)	77.7 (35.5)	89.6 (45.8)	90.4 (40.9)	70.5 (28.4)	72.4 (40.0)	78.0 (37.3)	79.0 (47.1)	89.2 (47.0)	104.9 (38.0)	73.1 (30.0)	69.7 (28.4)
Saturated fat (g)			32.3 (22.0)	32.2 (18.2)			24.2 (17.9)	24.8 (17.0)	32.1 (22.2)	33.9 (15.7)		
Polyunsaturated fat (g)			14.0 (9.4)	14.2 (8.0)					14.0 (8.7)	14.5 (10.6)		
Monounsaturated fat (g)			33.4 (18.3)	36.0 (20.4)					32.5 (17.8)	37.7 (15.8)		
Carbohydrate(g)	230.2 (82.2)	209.5 (97.8)	229.7 (90.6)	212.2 (99.3)	230.2 (80.0)	207.7 (101.3)	226.1 (87.4)	219.7 (88.6)	225.6 (93.1)	235.0 (124.9)	228.4 (84.7)	213.4 (78.9)
Sugars (g)	60.2 (46.6)	52.0 (61.7)	77.7 (47.4)	64.6 (89.6)	46.0 (41.0)	45.3 (34.8)	60.3 (45.7)	52.0 (51.0)	76.6 (58.2)	67.9 (55.1)	46.3 (42.7)	44.3 (23.0)
Alcohol (g)	0.0 (0.4)	0.0 (4.4)	0.02 (10.9)	0.5 (16.3)	0.0 (0.0)	0.0 (0.0)	0.0 (40.7)	0.0 (0.5)	0.02 (11.0)	0.1 (5.2)	0.0 (0.0)	0.0 (0.0)
Dietary Fibre (g)	15.9 (12.5)	14.7 (13.4)	22.2 (14.0)	21.4 (13.0)	8.6 (5.5)	10.7 (5.6)	16.0 (12.2)	12.1 (43.8)	22.0 (13.8)	24.9 (20.8)	10.6 (6.4)	8.6 (5.5)
Iron (mg)	10.1 (5.8)	9.8 (6.9)	11.0 (5.3)	11.2 (5.5)	7.9 (4.8)	8.9 (9.5)	10.2 (5.6)	8.8 (7.1)	11.1 (5.0)	11.8 (7.0)	8.6 (5.5)	7.2 (4.6)
**Total Additive Intake**	30.0 (22.8)	52.0 (29.3)	17.5 (70.0)	17.0 (30.0)	17.0 (18.0)	20.0 (30.0)	4.0 (22.5)	3.0 (30.5)	17.0 (68.0)	15.0 (41.0)	17.0 (19.0)	17.0 (30.0)
**Total Artificial Sweetener Intake**	720.0 (0.0)	0.0 (2.9)	0.0 (18.8)	0.0 (180.0)	0.0 (0.0)	0.0 (1.8)	0.0 (0.1)	0.0 (1.3)	0.0 (12.5)	0.0 (15.0)	0.0 (1.4)	0.0 (1.6)
**Total Emulsifier Intake**	0.0 (54.6)	0.0 (0.0)	0.0 (105.0)	0.0 (0.0)	0.0 (7.0)	0.0 (10.0)	0.0 (0.0)	0.0 (75.0)	0.0 (0.0)	0.0 (135.0)	0.0 (6.0)	0.0 (7.8)
Acesulfame K	480.0 (0.0)	0.0 (0.0)	0.0 (0.0)	0.0 (120.0)	0.0 (0.0)	0.0 (0.3)	0.0 (0.0)	0.0 (0.4)	0.0 (0.0)	0.0 (0.0)	0.0 (0.0)	0.0 (0.2)
Aluminosilicate	0.0 (0.0)	0.0 (0.0)	0.0 (0.0)	0.0 (0.0)	0.0 (0.0)	0.0 (0.0)	0.0 (0.0)	0.0 (0.0)	0.0 (0.0)	0.0 (0.0)	0.0 (0.0)	0.0 (0.0)
Aspartame	240.0 (0..0)	0.0 (0.0)	0.0 (0.0)	0.0 (60.0)	0.0 (0.0)	0.0 (0.0)	0.0 (0.0)	0.0 (0.0)	0.0 (0.0)	0.0 (7.5)	0.0 (0.0)	0.0 (7.5)
Carboxymethylcellulose	0.0 (0.0)	0.0 (0.0)	0.0 (0.0)	0.0 (0.0)	0.0 (0.0)	0.0 (10.0)	0.0 (0.0)	0.0 (75.0)	0.0 (0.0)	0.0 (75.0)	0.0 (0.0)	0.0 (0.0)
Carrageenan	0.0 (0.3)	0.0 (0.0)	0.0 (17.5)	0.0 (0.0)	0.0 (0.0)	0.0 (160.0)	0.0 (0.0)	0.0 (37.5)	0.0 (0.0)	0.0 (37.5)	0.0 (0.0)	0.0 (0.8)
Polysorbate-80	0.0 (0.0)	0.0 (0.0)	0.0 (0.0)	0.0 (0.0)	0.0 (0.0)	0.0 (0.0)	0.0 (0.0)	0.0 (0.0)	0.0 (0.0)	0.0 (0.0)	0.0 (0.0)	0.0 (0.0)
Saccharin	0.0 (0.0)	0.0 (0.0)	0.0 (0.0)	0.0 (180.0)	0.0 (0.0)	0.0 (0.0)	0.0 (0.0)	0.0 (0.0)	0.0 (0.0)	0.0 (0.0)	0.0 (0.0)	0.0 (0.0)
Sucralose	0.0 (0.0)	0.0 (0.0)	0.0 (0.0)	0.0 (0.0)	0.0 (0.0)	0.0 (0.0)	0.0 (0.0)	0.0 (0.0)	0.0 (0.0)	0.0 (0.0)	0.0 (0.0)	0.0 (0.0)
Sulphites	0.0 (0.0)	0.0 (0.0)	0.0 (320.0)	0.0 (0.0)	0.0 (0.0)	0.0 (0.0)	0.0 (0.6)	0.0 (1.0)	0.0 (0.6)	0.0 (1.0)	0.0 (0.0)	0.0 (0.0)
Titanium Dioxide	0.0 (0.0)	0.0 (0.0)	0.0 (0.0)	0.0 (0.0)	0.0 (0.0)	0.0 (0.0)	0.0 (0.0)	0.0 (0.0)	0.0 (0.0)	0.0 (0.0)	0.0 (0.0)	0.0 (0.0)
